# Proteomic profiles of peritoneal fluid-derived small extracellular vesicles correlate with patient outcome in ovarian cancer

**DOI:** 10.1172/JCI176161

**Published:** 2024-04-02

**Authors:** Miguel Quiralte, Arantzazu Barquín, Mónica Yagüe-Fernández, Paloma Navarro, Tatiana P. Grazioso, Elena Sevillano-Fernández, Juan F. Rodriguez-Moreno, Alejandra Balarezo-Saldivar, Héctor Peinado, Elena Izquierdo, Carlos Millán, Irene López-Carrasco, Mario Prieto, Rodrigo Madurga, Ismael Fernández-Miranda, Sergio Ruiz-Llorente, Jesús García-Donas

**Affiliations:** 1 Laboratory of Innovation in Oncology, Clara Campal Comprehensive Cancer Centre (HM CIOCC), HM Sanchinarro University Hospital, Madrid, Spain.; 2 Institute of Applied Molecular Medicine, Faculty of Medicine, Universidad San Pablo–CEU, Alcorcón, Madrid, Spain.; 3 HM CIOCC, HM Sanchinarro University Hospital, Madrid, Spain.; 4 Microenvironment and Metastasis Laboratory, Molecular Oncology Program, Spanish National Cancer Research Centre, Madrid, Spain.; 5 Gynecologic Unit, HM Montepríncipe University Hospital, Boadilla del Monte, Madrid, Spain.; 6 Department of Pathological Anatomy, Therapeutic Targets Laboratory, HM Sanchinarro University Hospital, Madrid, Spain.; 7 Faculty of Experimental Sciences, Francisco de Vitoria University, Pozuelo de Alarcón, Madrid, Spain.; 8 R&D Oncology Business Unit, Pharmacogenomic and Cell Biology Departments, PharmaMar, Colmenar Viejo, Madrid, Spain.; 9 Department of Biomedicine and Biotechnology, Genetics Area, Universidad de Alcalá, Alcalá de Henares, Madrid, Spain.

**Keywords:** Oncology, Cancer, Obstetrics/gynecology, Proteomics

## Abstract

Cancer-derived small extracellular vesicles (sEVs) are capable of modifying the tumor microenvironment and promoting tumor progression. Ovarian cancer (OvCa) is a lethal malignancy that preferentially spreads through the abdominal cavity. Thus, the secretion of such vesicles into the peritoneal fluid could be a determinant factor in the dissemination and behavior of this disease. We designed a prospective observational study to assess the impact of peritoneal fluid–derived sEVs (PFD-sEVs) in OvCa clinical outcome. For this purpose, 2 patient cohorts were enrolled: patients with OvCa who underwent a diagnostic or cytoreductive surgery and nononcological patients, who underwent abdominal surgery for benign gynecological conditions and acted as the control group. Systematic extraction of PFD-sEVs from surgical samples enabled us to observe significant quantitative and qualitative differences associated with cancer diagnosis, disease stage, and platinum chemosensitivity. Proteomic profiling of PFD-sEVs led to the identification of molecular pathways and proteins of interest and to the biological validation of S100A4 and STX5. In addition, unsupervised analysis of PFD-sEV proteomic profiles in high-grade serous ovarian carcinomas (HGSOCs) revealed 2 clusters with different outcomes in terms of overall survival. In conclusion, comprehensive characterization of PFD-sEV content provided a prognostic value with potential implications in HGSOC clinical management.

## Abstract 2

High-grade serous ovarian carcinomas outcome correlates with the proteomic cargo of small extracellular vesicles extracted from the peritoneal cavity.

## Introduction

Ovarian cancer (OvCa) is the fifth leading cause of mortality among women and the foremost cause of death attributed to gynecological cancers in developed countries (1). Patients with OvCa present high recurrence rates within 16 to 22 months after completing radical surgery followed by conventional platinum-based chemotherapy (2). While numerous studies have assessed physical pelvic examination, transvaginal ultrasound, and serum carbohydrate antigen 125 (CA125) as screening methods, these procedures have shown low sensitivity and specificity (3–5). Thus, the majority of cases are diagnosed at advanced stages, when the tumor has typically disseminated throughout the abdominal cavity (6). Consequently, there is an urgent need to elucidate the molecular mechanisms triggering OvCa metastasis, identify predictive biomarkers, and discover novel targets to enhance therapeutic efficiency (7).

In the last decade, several studies have highlighted the role of extracellular vesicles (EVs) in the pathogenesis of multiple human diseases (8, 9). Small extracellular vesicles (sEVs) represent a subtype of EVs smaller than 200 nm that originate from multivesicular endosomes. These sEVs encapsulate a wide variety of biomolecules (including nucleic acids, proteins, and lipids, among others) that are secreted into the extracellular space and can modulate the physiology of target cells that internalize them (10). In the context of cancer, tumor-secreted sEVs contain molecules that are able to promote angiogenesis, facilitate intercellular communication between tumor cells and their microenvironment, modulate the immune response, and remodel surrounding and distal tissues. Consequently, sEVs could favor tumor progression through the establishment of a premetastatic niche (8, 11), the development of therapeutic resistance through a crosstalk between tumor cells, and immune evasion through the manipulation of immune cells (12, 13).

From a clinical perspective, the characterization of the cargo of sEVs secreted by the tumor could be relevant for the identification of diagnostic, predictive, and prognostic markers (8). Prior studies have isolated sEVs from peripheral blood and peritoneal fluids of patients with OvCa to identify diagnostic miRNA signatures (14–16). The protein content of OvCa-derived sEVs has been evaluated to a lesser extent and has been limited to the screening of specific proteins or the use of low-range customized panels for diagnostic purposes (16). However, these proteins may serve as the true effectors of most of the functions attributed to sEVs. Thus, high-throughput proteomic analysis could help to determine and understand the role of sEVs and their cargo proteins in the outcomes of patients with OvCa.

The trans-coelomic route is the most frequent form of OvCa metastatic dissemination, characterized by peritoneal spread and the formation of malignant ascites (17). Therefore, peritoneal fluid could act as a reservoir comprising multiple cellular components and soluble factors composing the characteristic microenvironment of this tumor (15). Though massive ascites is a common event occurring in the latter steps of the disease, obtaining samples for proper analysis at the initial diagnosis stage is challenging (18). Considering these premises, we systematically collected peritoneal washings and malignant ascites during the surgical procedures of 65 patients with OvCa throughout the clinical course of their disease, as well as from 29 women who underwent abdominal interventions due to nonmalignant conditions. The experimental approach subsequently included (a) the comparison of mass spectrometry proteomic profiling of the sEV cargo in the OvCa cohort versus the nononcological patient cohort, (b) data integration with detailed clinical information available for patients with OvCa, and (c) the biological validation of differentially sEV-contained proteins through immunoblotting. Our work provides an essential proof of concept that the study of the protein cargo of peritoneal fluid–derived sEVs (PFD-sEVs) represents an efficient strategy for the identification of prognostic signatures in OvCa.

## Results

### Clinical features and molecular characterization of OvCa and nononcological patients.

A prospective observational study was designed to recruit patients with OvCa and nononcological gynecological conditions scheduled for abdominal surgery as part of the standard management. This study involved the participation of 4 health institutions in Madrid, Spain, which collaborated in the recruitment of participants.

From June 2018 to September 2022, a total of 74 peritoneal fluids derived from 65 patients with OvCa were collected. This cohort included serial samples from 8 patients, which comprised diagnostic laparoscopies and interval surgeries, with a third sample collected for one of these patients from a salvage surgery (in recurrence). Histological subtypes included high-grade serous ovarian carcinoma (HGSOC; 54 cases [83.1%]), endometrioid carcinoma (6 [9.2%]), low-grade serous (2 [3.1%]), clear cell (2 [3.1%]), and mucinous (1 [1.5%]) (Table 1). Following the FIGO classification (19), tumor stages reported at the time of surgery included stage I–II in 7 patients (10.8%), stage III in 38 (58.4%), and stage IV in 20 (30.8%) (Table 1). Next-generation sequencing panels (FoundationOne CDx [Foundation Medicine] and/or iD.BRCA [AstraZeneca] test) were performed as routine practice in 54 patients, revealing *BRCA1/2* alterations in 16 patients (30%). Of these, 7 showed germline mutation (43.75%), 6 presented somatic mutation (37.5%), and the origin of the mutation could not be confirmed in 3 cases (Table 1). Detailed definitions pertaining to clinicopathological and molecular criteria can be found in Methods.

In parallel, 29 nononcological patients were recruited to be part of the control cohort (hereafter referred to as controls). The pathological diagnoses included serous (*n* = 7 [24.1%]) or mucinous (*n* = 3 [10.35%]) ovarian cystadenomas, uterine myomas (*n* = 7 [24.1%]), ovarian cystic teratomas (*n* = 2 [6.9%]), endometriosis (*n* = 4 [13.8%]), normal tissue (prophylactic surgeries) (*n* = 2 [6.9%]), uterine prolapse (*n* = 3 [10.35%]), and endometrial hyperplasia (*n* = 1 [3.5%]) (Table 1).

### PFD-sEV concentration correlates with tumor stage and disease progression.

**Figure 1:**
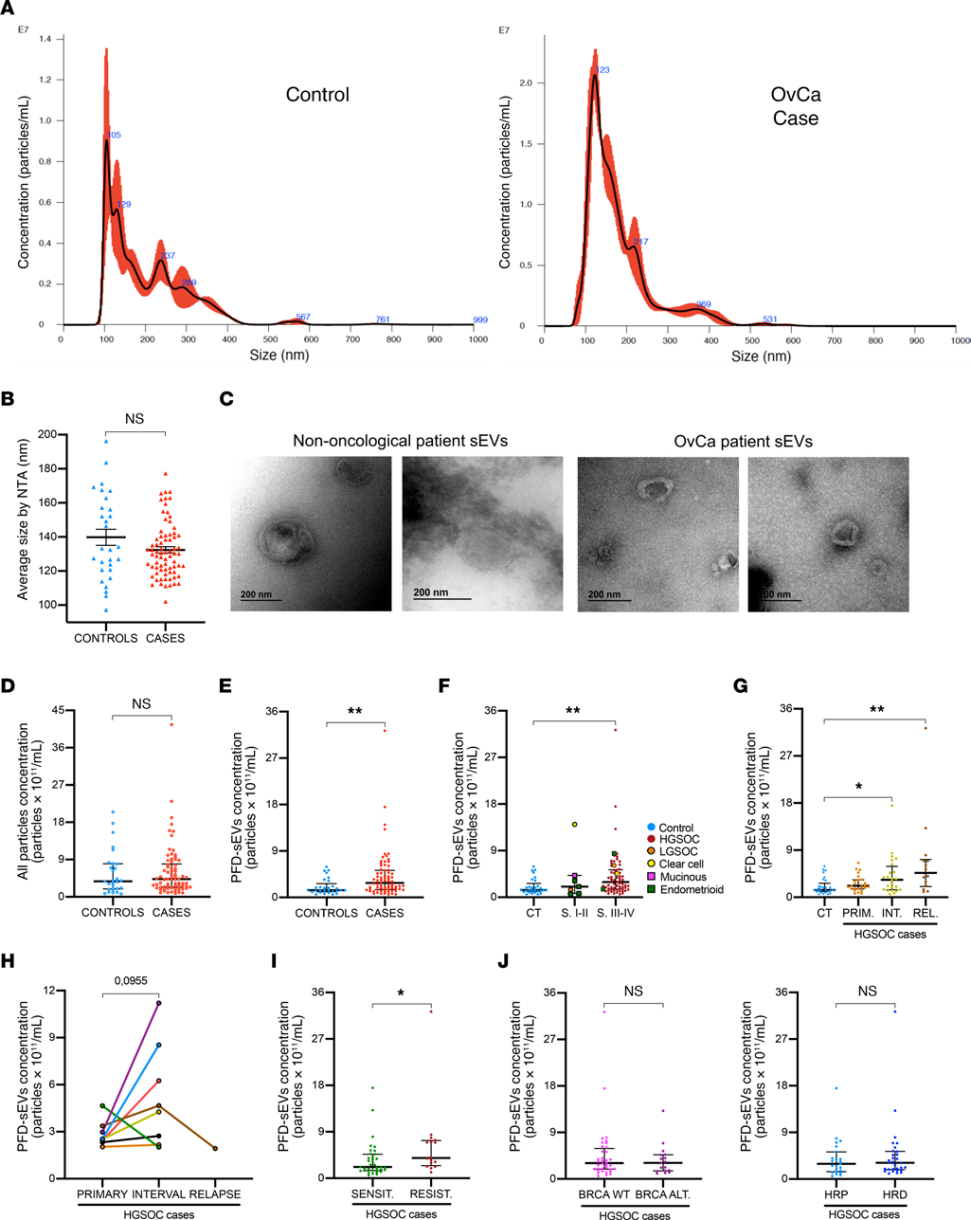
Characterization of PFD-sEVs in controls and patients with OvCa. (**A**) Representative image of particle size distribution determined by NTA in control and OvCa patient samples. (**B**) Primary peak size of particles measured by NTA in control (*n* = 29) and OvCa patient samples (*n* = 74). (**C**) EV morphology observed by transmission electron microscopy in controls (*n* = 2) and patients with OvCa (*n* = 2). Scale bars: 200 nm. (**D**) Concentration analysis of all particles in control (*n* = 29) and OvCa patient samples (*n* = 74). (**E**) Analysis of PFD-sEV concentration in control (*n* = 29) and OvCa patient samples (*n* = 74). (**F**) Analysis of PFD-sEV concentration in control (CT, *n* = 29) and OvCa patient samples (*n* = 74) separated according to tumor stage (S) (stage I–II, *n* = 7; stage III–IV, *n* = 67). Additional information regarding histology is provided (HGSOC, high-grade serous ovarian carcinomas; LGSOC, low-grade serous ovarian carcinomas). (**G**) Analysis of PFD-sEV concentration in control (*n* = 29) and HGSOC patient samples according to surgical origin of samples (PRIM, primary/diagnostic [*n* = 25]; INT, interval [*n* = 24]; REL, relapse [*n* = 14]). (**H**) PFD-sEV concentration at different time points from HGSOC cases with serial samples (*n* = 8). (**I**) PFD-sEV concentration according to cisplatin sensitivity (SENSIT, sensitive [*n* = 35]; RESIST, resistant [*n* = 16]) in HGSOC cases. (**J**) PFD-sEV concentration in HGSOC cases according to *BRCA* status (WT, wild type [*n* = 36]; ALT, altered [*n* = 17]) or HRD status (HRP, homologous recombination proficient [*n* = 23]; HRD, homologous recombination deficient [*n* = 30]). Unless otherwise indicated, data are shown as median and IQR from each independent sample/experiment. *P < 0.05, **P < 0.01, Mann-Whitney test (**B**, **D**, **E**, **I**, and **J**) or Kruskal-Wallis test with Dunn’s multiple-comparison test and Bonferroni’s adjusted *P* values (**F** and **G**).

Peritoneal fluid ultracentrifugation was used to extract sEVs from patients with OvCa (*n* = 74 samples from 65 patients) and control patients (*n* = 29). Vesicle size analysis by nanoparticle tracking analysis (NTA) in both cohorts showed an efficient and homogeneous collection of particles in the range of sEVs (<200 nm; hereafter referred to as PFD-sEVs) (Figure 1A) with similar average primary peak size for both sample sets (OvCa cases, 132.5 nm, vs. controls, 139.8 nm) (Figure 1B). In accordance, transmission electron microscopy analysis showed the presence of similar round-shaped vesicles in the sEV size range (<200 nm) in samples from both patients with OvCa and controls (Figure 1C). NTA profiling additionally showed that there was no significant difference in the number of peritoneal fluid–derived particles obtained from patients with OvCa compared with controls (Figure 1D). However, a significantly higher concentration was observed in OvCa cases when the size range was restricted to that associated with PFD-sEVs (OvCa cases, 2.81 × 10^11^ particles/mL, vs. controls, 1.41 × 10^11^ particles/mL; *P <* 0.01) (Figure 1E).

When OvCa cases were subdivided according to tumor stage, differences between patients with OvCa and controls were shown to be mainly determined by a higher number of vesicles detected in patients with stages III–IV (Figure 1F). Notably, the HGSOC subtype was the only histology that showed statistically significant differences compared with controls. However, it is important to highlight that the number of OvCa cases with non-high-grade serous histology was limited, and the majority of them were diagnosed as stage I or II tumors (Supplemental Figure 1A; supplemental material available online with this article; https://doi.org/10.1172/JCI176161DS1). Thus, subsequent analyses concerning the number of PFD-sEVs were restricted only to patients with HGSOC, the most frequently diagnosed histology both in clinical practice and within our study.

HGSOC-related analysis of particle concentration according to the surgical origin of the sample showed that the vesicular content was significantly higher upon relapse or after neoadjuvant chemotherapy (interval surgery) in comparison with chemotherapy-naive samples (Figure 1G). Accordingly, when serial samples from patients were analyzed (*n* = 8), the particle concentration tended to increase at interval surgeries (performed after neoadjuvant chemotherapy) when compared with diagnostic procedures (chemo-naive samples) (2.0-fold change on average at interval) (Figure 1H). Regarding HGSOC platinum-based chemotherapy response, platinum-resistant cases showed a significantly higher PFD-sEV concentration than platinum-sensitive cases (Figure 1I). However, no statistically significant differences in the concentration of sEVs were observed when patients with HGSOC were classified according to the presence of BRCA1/2 alterations or homologous recombination (HR) status (Figure 1J). Finally, given that the presence of residual disease after cytoreductive surgery is one of the major predictive factors in OvCa, we compared the quantity of sEVs in patients with HGSOC who achieved an R0 resection versus R1, and no significant differences were observed (Supplemental Figure 1B). Likewise, a lack of correlation was observed between the concentration of sEVs and the source of PFD-sEVs (peritoneal washings vs. ascites) (Supplemental Figure 1C).

### PFD-sEV protein concentration is associated with HGSOC-related clinical features.

**Figure 2:**
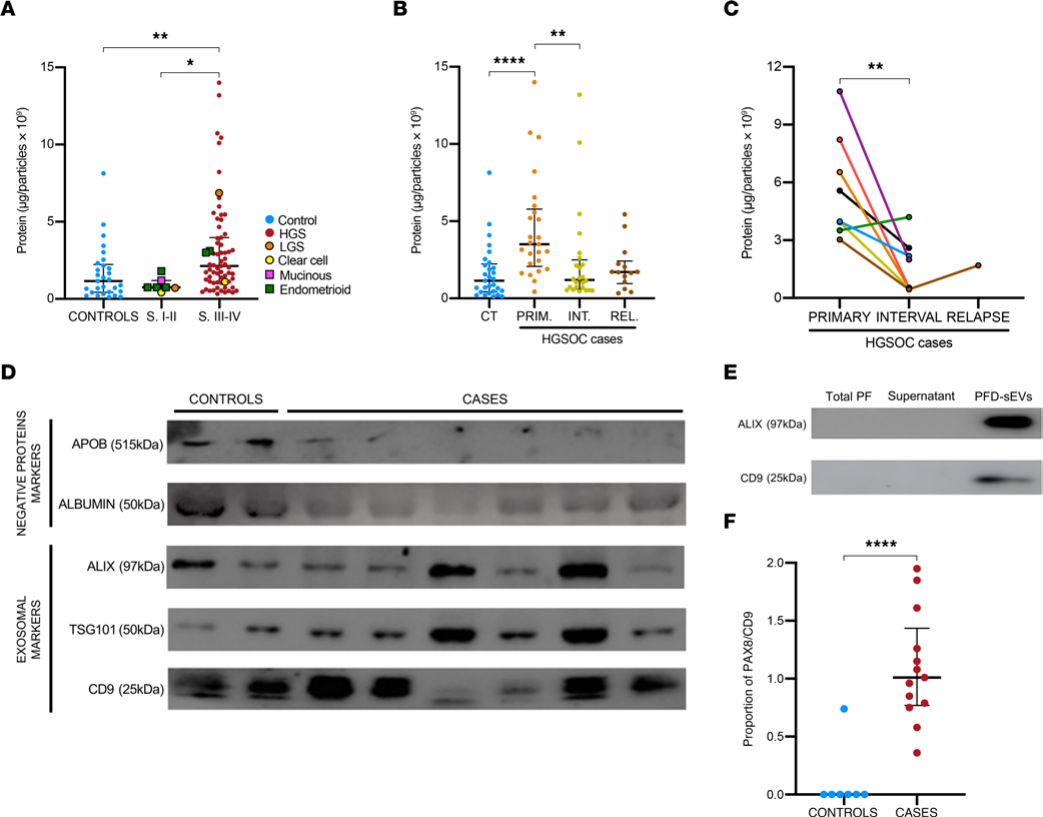
Quantitative analysis of protein content per particle per 10^9^ in PFD-sEVs. (**A**) Ratio of protein per particle in control (*n* = 29) and OvCa patient samples separated according to tumor stage (stage I–II, *n* = 7; stage III–IV, *n* = 67). Histological subtype information regarding each tumor is also provided. (**B**) Ratio of protein per particle in control (CT, *n* = 29) and HGSOC patient samples according to surgical origin of the sample (PRIM, primary/diagnostic [*n* = 25]; INT, interval [*n* = 24]; REL, relapse [*n* = 14]). (**C**) Evolution of normalized protein levels regarding the PFD-sEV concentration in the different serial samples from patients with HGSOC (*n* = 8). (**D**) Representative Western blot of EV markers (ALIX, TSG101, and CD9) and markers of non-EV co-isolated structures (APOB and albumin) in a selected set of controls and OvCa samples. (**E**) Representative Western blotting analysis of EV markers including ALIX and CD9 was performed in OvCa cases at different stages during the sEV isolation process. Total peritoneal fluid was collected before the start of the isolation process. Supernatant sample was collected immediately after the first ultracentrifugation step, and “PFD-sEVs” corresponds to the pellet obtained after the ultracentrifugation steps. (**F**) Quantification of normalized PAX8/CD9 ratios obtained through immunoblotting in control (*n* = 7) and OvCa patient samples (*n* = 13). Unless otherwise indicated, data are shown as median and IQR from each independent sample/experiment. *P < 0.05, **P < 0.01, ****P < 0.0001, Kruskal-Wallis test with Dunn’s multiple-comparison test and Bonferroni’s adjusted *P* values (**A** and **B**), paired 2-tailed *t* test (**C**), or Mann-Whitney test (**F**).

PFD-sEV protein content was quantified through bicinchoninic acid assay. Protein content per particle (PCP) in PFD-sEVs was significantly higher (2.0- to 3.0-fold change) in OvCa cases diagnosed at stages III–IV (2.13 μg per 1 × 10^9^ particles) when compared with both controls (1.15 μg per 1 × 10^9^ particles) and low-stage tumors (0.75 μg per 1 × 10^9^ particles) (*P* < 0.01 and *P* < 0.05, respectively) (Figure 2A). It is worth mentioning that the values corresponding to stages III–IV are 10 times higher than those observed by other authors in sEVs obtained from control patient serum (20), which may be related to an active secretory behavior of ovarian carcinoma and its tumor microenvironment at advanced stages. Similar to the quantitative data related to the number of vesicles (Figure 1B), we also detected significant differences in protein concentration when comparing HGSOC cases with controls, but no differences were observed for the remaining histological subtypes (Supplemental Figure 2A). However, as previously mentioned, non-HGSOC patients were poorly represented in our study and most cases were diagnosed at early stages, which could be a key confounding factor.

We subsequently analyzed PCP considering exclusively HGSOC histology. When these patients were classified based on surgical origin of the sample, it was noted that sEVs obtained from primary surgeries showed a higher PCP (3.5 μg per 1 × 10^9^ particles) than those acquired from interval (1.19 μg per 1 × 10^9^ particles) or relapse surgeries (1.71 μg per 1 × 10^9^ particles) (*P* < 0.001 and *P* < 0.01, respectively) (Figure 2B). A similar trend was observed when comparing PCP from serial samples obtained from different patients with HGSOC, particularly between diagnostic samples and interval surgery (6.5-fold change in concentration on average at diagnosis, *P* < 0.01) (Figure 2C).

Given the patterns observed in HGSOC with respect to vesicle concentration (Figure 1G) and PCP (Figure 2B), we analyzed the potential association between these 2 variables through Pearson’s regression analysis and observed a significant inverse correlation (Supplemental Figure 2B). While interval and relapse surgeries showed a heterogeneous behavior, primary surgery samples were homogeneously characterized by a reduced number of vesicles but increased protein content. Finally, while no statistically significant differences in PCP were noted when patients with HGSOC were grouped by R0 versus R1 status (Supplemental Figure 2C), categorization of cases based on the sample source revealed a significant PCP increase in ascites versus peritoneal washings (Supplemental Figure 2D).

### PDF-sEVs contain protein markers associated with the development of OvCa.

The purity of sEV preparations was confirmed through the analysis of exosomal markers (ALIX, TSG101, CD9) and negative protein markers (APOB and albumin), in compliance with the Minimal Information for Studies of Extracellular Vesicles (MISEV) 2023 guidelines (21), in a representative subset of PFD-sEV samples obtained from patients with OvCa or controls (Figure 2D). Moreover, immunoblotting profiling of the different supernatants or fractions collected throughout the ultracentrifugation-mediated extraction showed a robust expression of markers associated with exosome biogenesis (ALIX) or membrane dynamics and morphology (CD9 tetraspanin) exclusively in the PFD-sEV suspension (Figure 2E). On the contrary, supernatants obtained in previous steps of the PFD-sEV isolation (total peritoneal fluid or supernatant) displayed an absolute absence of expression of these markers (Figure 2E), denoting a selective and efficient extraction of PFD-sEVs.

To determine whether the PFD-sEV fraction isolated from oncological patients contained vesicles specifically secreted by the ovarian tumor cells, we evaluated through immunoblotting the expression of the paired box 8 (PAX8) protein in a subset of OvCa cases (*n* = 13) and controls (*n* = 7). Despite the fact that PAX8 is mostly known for codifying a transcription factor essential in the physiology of thyroid follicular cells, its overexpression has been widely described in the context of ovarian carcinomas, representing a reliable and widely used diagnostic marker for gynecological pathologies derived from the fallopian tube secretory epithelial cells (22). As depicted in Figure 2F, all OvCa cases showed PAX8 expression in PFD-sEV samples, whereas only 1 of the 7 control samples tested showed positivity for this factor (normalized PAX8/CD9 ratio, *P* < 0.001) (Figure 2F and Supplemental Figure 2E). These findings confirmed that purified PFD-sEVs from patients with OvCa were secreted by ovarian carcinoma cells, prompting us to perform mass spectrometry profiling to identify protein biomarkers related to disease outcome, define molecular pathways modulated by tumor sEVs, and better classify HGSOCs on the basis of their PFD-sEV proteomic patterns.

### PFD-sEV proteomics revealed a differential cargo of ovarian carcinoma–related biomarkers in patient cohort.

The selection of OvCa cases and controls to be analyzed by mass spectrometry was performed considering the amount of total protein required for proteomic profiling (≥20 μg). The protein cargo of PFD-sEVs was profiled by liquid chromatography with tandem mass spectrometry (LC-MS/MS) in samples from 29 patients with OvCa and 10 controls.

**Figure 3:**
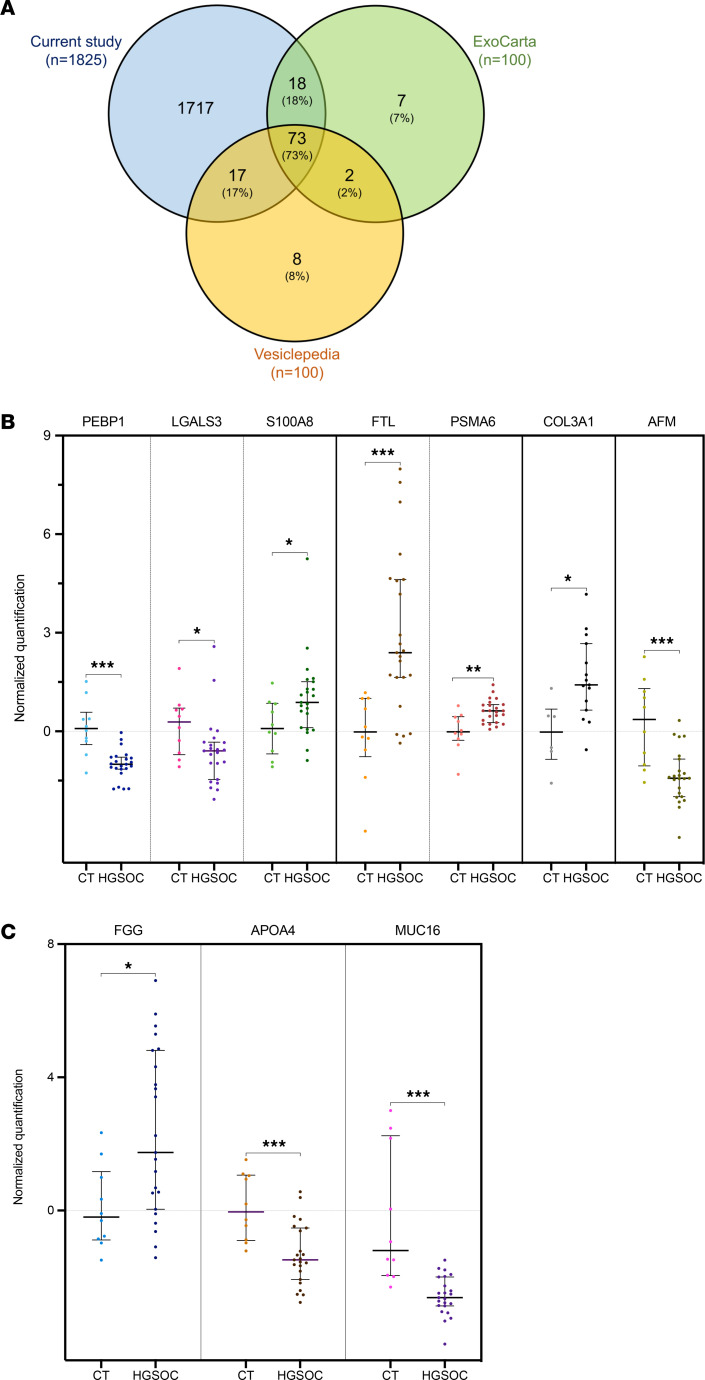
Comparison of sEV-related protein cargo with sEV biomarker databases and OvCa proteomic profiling from previous studies. (**A**) Venn diagram showing the overlap between the proteins identified in PFD-sEVs in the current study and the top 100 proteins from the Vesiclepedia and ExoCarta databases. (**B**) Graph depicting the proteomic normalized quantification data for 7 proteins previously described as protein OvCa biomarkers. Comparison is established between controls (CT, *n* = 10) and HGSOC cases (*n* = 23). *P < 0.05, **P < 0.01, ***P < 0.001, Mann-Whitney test. Dark vertical lines separate markers previously described by 4 independent studies (23). (**C**) Graphs showing normalized ratios of FGG, APOA4, and MUC16 protein expression in PFD-sEVs comparing samples from our discovery cohort (patients with HGSOC [*n* = 23] vs. controls [*n* = 10]). These factors have previously been described as diagnostic markers for OvCa in serum-derived sEVs (16). Benjamini-Hochberg adjusted *P* values: *P < 0.05, ***P < 0.001, Mann-Whitney test.

LC-MS/MS proteomic characterization of the PFD-sEV cargo allowed the identification of 20,899 peptides (FDR < 1%, calculated at peptide level) corresponding to 1,825 proteins (Supplemental Data 1). The list of proteins identified in the discovery cohort was compared against a reference list of exosome and EV markers obtained from reference repositories (Vesiclepedia and ExoCarta). As a result, we were able to detect the presence of more than 90% of the 100 most frequently listed proteins in both databases within our study samples, including the well-known sEV markers CD9, CD63, and CD81 (Figure 3A). These findings, together with those related to the qualitative analysis of vesicle size by NTA, denoted that sEVs were efficiently isolated by the experimental approach used in our study.

PFD-sEV proteomic data from HGSOC cases were further compared with previously published studies focused on identifying differentially expressed proteins between different specimens of OvCa cases versus their corresponding normal counterparts (Supplemental Figure 3A) (23). Seven proteins (PEBP1, LGALS3, S100A8, FTL, PSMA6, COL3A1, and AFM) showed significant changes (*P* < 0.05) and a similar expression trend in our proteomic data when compared with the previously mentioned study (23) (Figure 3B). Moreover, additional biomarkers presented a similar trend in terms of PFD-sEV cargo when compared with the data previously described (Supplemental Figure 3B) (23). In accordance with the results obtained by Lai et al. (16) by means of protein profiling in peripheral blood–derived exosomes, our data also confirmed the diagnostic value of FGG and APOA4, but not of MUC16 (which showed an opposite expression ratio) (Figure 3C). Paralleling the conclusion drawn from the PAX8 immunoblotting results, the robust correlation with these OvCa markers may suggest that our PFD-sEV extracts represent biological specimens of interest for the study of the mechanisms involved in the progression and prognosis of HGSOC.

### Proteomic cargo differs depending on disease status and correlates with HGSOC overall survival.

**Figure 4:**
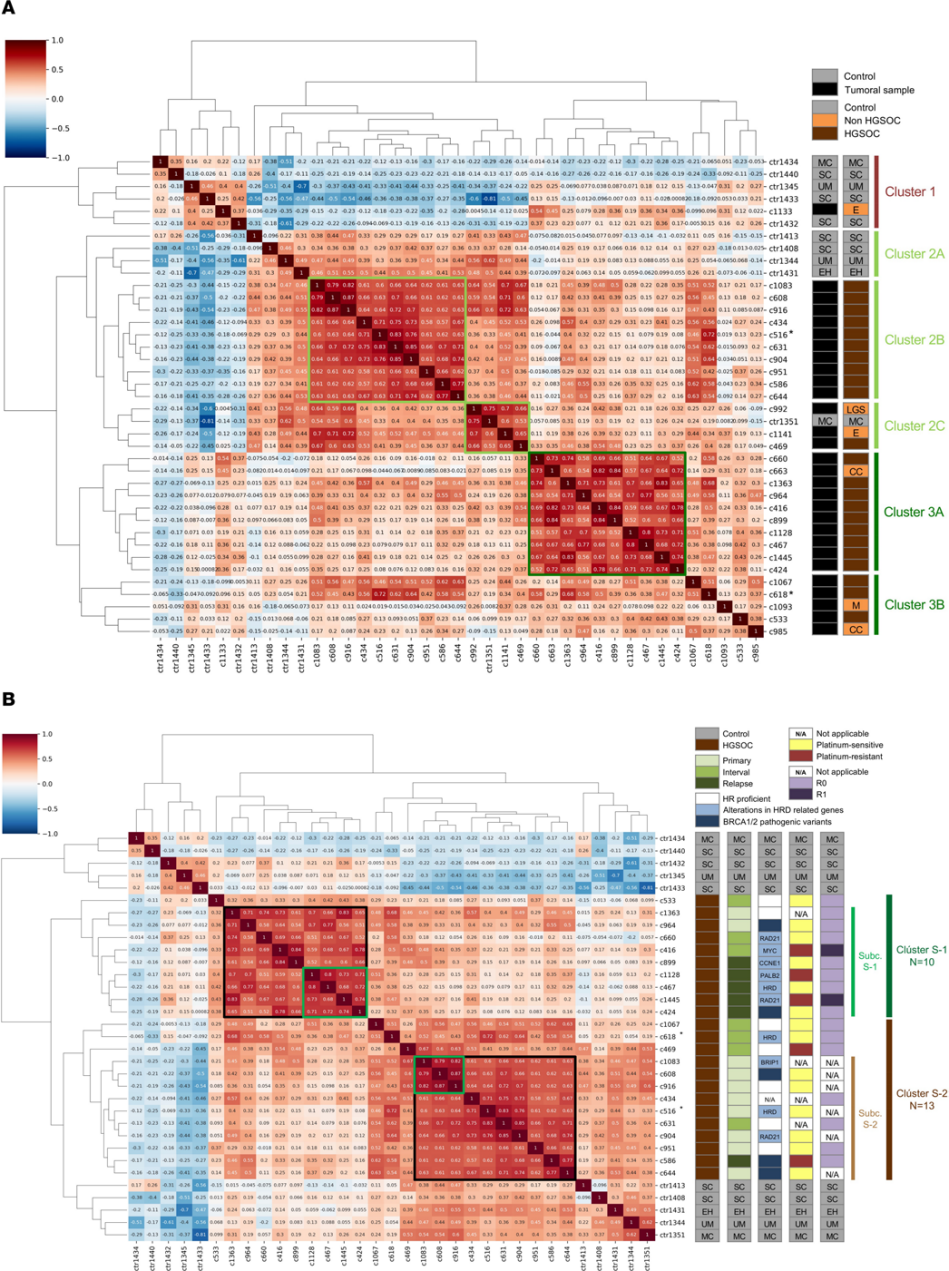
Correlation clustering map depicting the unsupervised analysis for the PFD-EV proteins profiled by mass spectrometry. (**A**) Unsupervised analysis for all cases and controls. At right, the main clusters (1, 2, and 3) and subclusters (2A–C and 3A–B) and the key clinical characteristics of the samples being compared are highlighted (controls [SC, serous ovarian cystadenoma; MC, mucinous ovarian cystadenoma; UM, uterine myoma; EH, endometrial hyperplasia] vs. OvCa tumoral sample; HGSOC vs. non-HGSOC [E, endometrioid; LGS, low-grade serous; M, mucinous; CC, clear cell]). Correlation clusters with highest ratios are labeled in light or dark green boxes. (**B**) Unsupervised analysis for HGSOC cases and controls. At right, the main carcinoma clusters (S-1, *n* = 10; and S-2, *n* = 13) and the key clinical characteristics of the samples being compared are highlighted (controls [SC, MC, UM, and EH] vs. HGSOC; surgical procedures: primary, interval, or recurrence surgeries; HRD status: BRCA1/2 pathogenic variants, alterations in HRD-related genes, HR proficient, or not applicable [NA]; platinum sensitivity: platinum-sensitive, platinum-resistant, or NA; surgical outcome: R0, R1, or NA). Main correlation subclusters for both S-1 and S-2 are depicted in dark green boxes, which are mainly constituted by 55% relapses (subc. S-1, 5/9) or 80% diagnostic/primary specimens (subc. S-2, 8/10). Light green boxes include the highest correlated samples and are associated exclusively with relapses (average correlation, 0.76) or diagnostic samples (average correlation, 0.82). The color bar on the left indicates the degree of correlation between 2 samples under study, with a value of 1 (dark red) indicating an identical sample in terms of protein cargo and –1 (dark blue) indicating potential samples with completely opposite profiles. Asterisks denote serial samples (C516 and C618) from one HGSOC case.

We then conducted an unsupervised clustering map based on the correlation of the 1,825 proteins identified by mass spectrometry. The inclusion of samples from all patients with OvCa and controls confirmed a separation of nontumor specimens into 2 clusters with different expression profiles (clusters 1 and 2A), which included an OvCa sample (C1133). Interestingly, this patient presented an early-stage, low-grade endometrioid tumor, and underwent a microscopically margin-negative resection (R0) without the need for chemotherapy. With respect to the tumor samples, OvCa cases were subclassified into 2 well-differentiated clusters (clusters 2B–C and 3A–B) (Figure 4A and Supplemental Data 1). In both clusters, we observed an indistinct distribution of HGSOC in 2 additional sets (subclusters 2B and 3A) and the preferential accumulation of non-HGSOC samples in specific branches (subclusters 2C and 3B). Subcluster 2C included one patient who at the time of diagnosis had a mucinous cystadenoma (ctrl1351).

Next, we exclusively selected HGSOC cases, which represented the most frequent histology in our study, along with controls for further analysis (Figure 4B and Supplemental Data 1). The cluster map exhibited a clear separation between cases and controls, and 2 main clusters of OvCa cases (S-1 and S-2) were clearly distinguishable. The principal S-1 subcluster (subc. S-1) preferentially gathered samples obtained in surgeries from tumor recurrences (5/9 OvCa cases, 55%). These samples also clustered together in a specific subset with a robust correlation ratio (C1128, C467, C1445, and C424; correlation ratio average 0.76). In contrast, the main S-2 subcluster (subc. S-2) preferentially included diagnostic and primary (chemo-naive) samples (8/10, 80%) and showed a subset of samples with the highest correlation rate among the entire analysis (C1083, C608, and C916; correlation ratio average 0.82) harboring exclusively diagnostic specimens. Regarding the samples obtained from interval debulking surgeries, although their distribution was uneven in the clustering map, there was a tendency toward clustering close to each other (C1067, C618, and C469 or C660 and C416). It is worth mentioning that paired samples (C618, interval, and C516, diagnostic) belonging to the same patient showed a high correlation in their vesicle content (correlation index 0.72) and were grouped with samples obtained at similar surgical time points instead of being clustered together. Similar clustering results were obtained both for the comparison between recurrence and interval or primary surgery (Supplemental Figure 4, A and B, and Supplemental Data 1) and for the proteomic data–based principal component analysis (PCA) (Supplemental Figure 4C and Supplemental Data 1), which exclusively considered proteins detected for all the samples under study. These findings were corroborated by hierarchical clustering, since ConsensusClusterPlus package analysis revealed a stable structure based on 5 clusters being able to differentiate 2 main clusters constituted by HGSOC samples (Supplemental Figure 4D and Supplemental Data 1).

**Figure 5:**
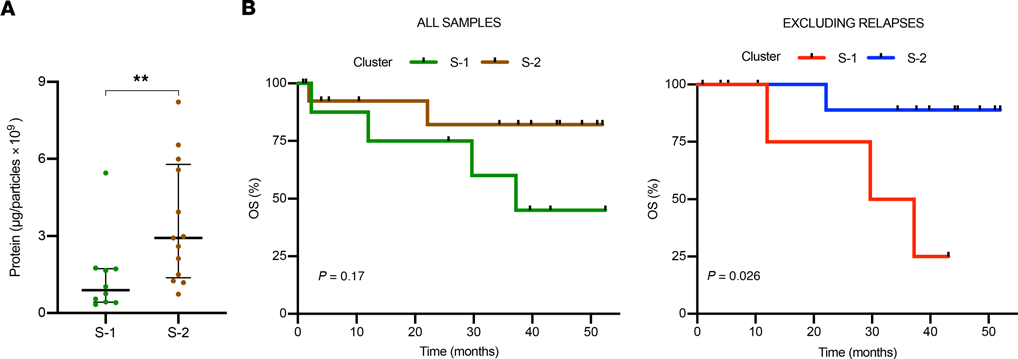
Correlation of proteomics-defined HGSOC main clusters (S-1 vs. S-2) with quantitative results of sEVs or with relevant clinical variables. (**A**) Association of clusters S-1 and S-2 with protein content per particle. Data are shown as median and IQR from each independent sample/experiment. **P < 0.01, Mann-Whitney test. (**B**) Association of proteomics-described HGSOC clusters with overall survival (months) either including all samples (left; S-1 [*n* = 10] vs. S-2 [*n* = 13]) or excluding those patients with recurrent disease (right; S-1 [*n* = 5] vs. S-2 [*n* = 12]).

Considering the 2 main sets of correlations obtained from the proteomic data of the 23 HGSOC samples, significant differences were observed in terms of protein concentration (Figure 5A), which could denote a different secretory behavior depending on the disease status. Regarding patient outcome, cluster S-1 (*n* = 10) presented a tendency toward a shorter overall survival (OS) median (37.2 months) than S-2 (*n* = 13) (not reached). Median overall follow-up was 34.4 months (27.7 months for the S-1 cluster vs. 37.6 for the S-2 cluster) (Figure 5B). When comparison was restricted to patients with HGSOC whose samples were obtained by the time of the initial therapeutic intervention (diagnostic/primary and interval surgeries), this difference became significant (S-1, *n* = 5, vs. S-2, *n* = 12; median overall follow-up: 37.2 months [29.7 for S-1 cluster vs. 38.7 for S-2 cluster], *P* < 0.05) (Figure 5B). Furthermore, despite the limited sample size and follow-up duration, significant differences in OS were observed when comparative analysis between S-1 and S-2 was additionally restricted to patients undergoing primary or interval surgeries and harboring defects in HR, a molecular factor of relevance in the prognosis of HGSOC (Supplemental Figure 5). Globally, these results denote that the vesicle cargo associates not only with HGSOC disease status and stage but also with its clinical outcome.

### Proteomics-based clusters of HGSOC cases are not associated with BRCA alterations, HR status, or sensitivity to chemotherapy.

It is widely established that around 20% of HGSOC cases harbor germline or somatic *BRCA* pathogenic alterations. However, this percentage rises to 50% when mutations in other susceptibility genes associated with homologous recombination deficiency (HRD) are considered (19). Taking into account the genetic background of our HGSOC cases, pathogenic alterations impairing *BRCA1/2* or other HRD-related genes (such as *PALB2*, *BRIP1*, *RAD21*, *CCNE1* amplification) and HRD scores were similarly distributed among the proteomic clusters S-2 and S-1 (7/12 [58%] vs. 8/10 [80%], respectively) (Supplemental Figure 6A and Supplemental Data 1). Moreover, platinum-based sensitivity did not present any differences between the clusters (Supplemental Figure 6B and Supplemental Data 1). Thus, *BRCA*/HRD status and chemosensitivity, two well-established prognostic factors, did not present differences in distribution among PFD-sEV proteomic clusters. This favors the hypothesis that the statistically significant difference in OS between S-1 and S-2 clusters could be attributed to their PFD-sEV proteomic profiles rather than to any other confounding factor, especially when the comparison was restricted to nonrelapsed samples. This restriction would avoid the selection bias of HGSOC cases that are considered suitable for a surgery at relapse (typically in patients with low-volume disease, good performance status, and long prior platinum-free interval), and the biological alterations of the PFD-sEV proteomic profiles induced by the exposure to chemotherapy (as previously shown). Unfortunately, the small numbers in our study preclude a multivariate analysis to definitively answer this intriguing question.

### Profiling of PFD-sEV cargo proteins reveals factors associated with specific HGSOC clinical variables.

**Figure 6:**
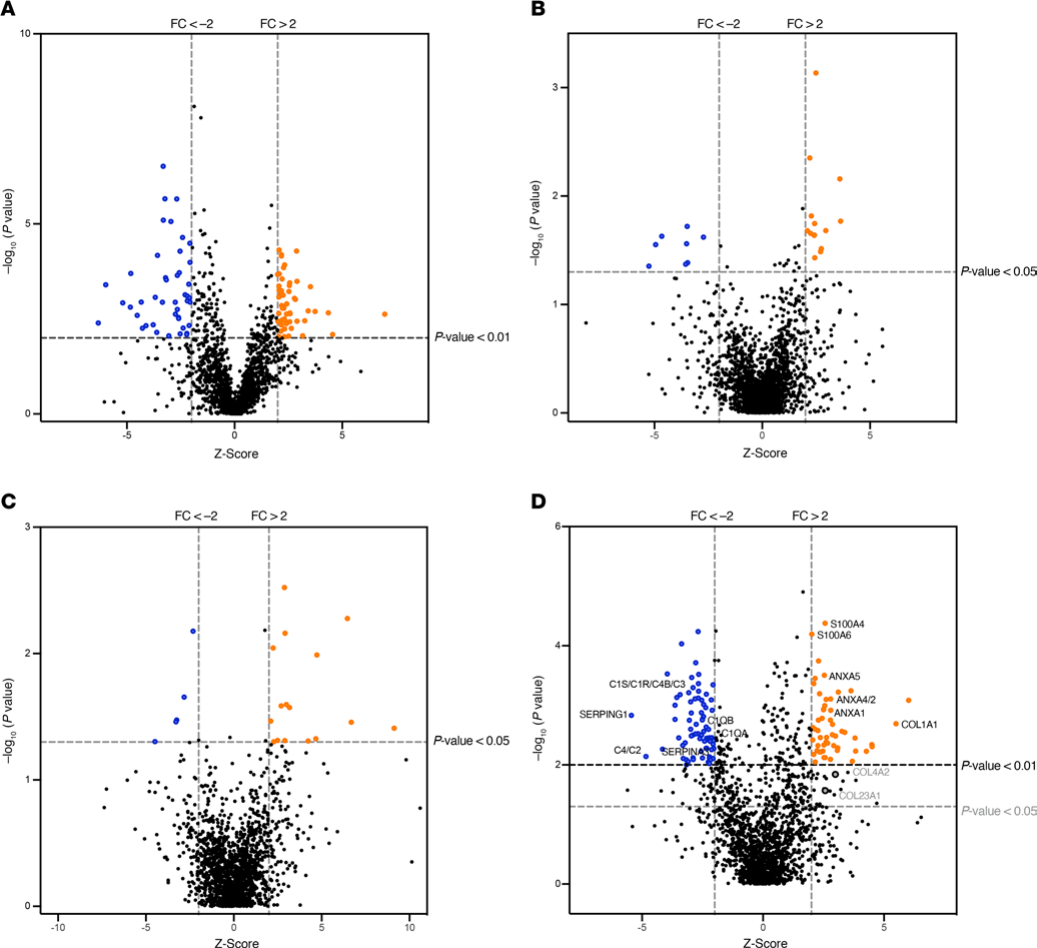
Volcano plots depicting differentially deregulated proteins in comparisons of interest. (**A**) HGSOC versus controls (deregulated proteins [DEPs] = 96). (**B**) Cisplatin-resistant versus -sensitive patients (DEPs = 22). (**C**) BRCA-altered vs. WT (DEPs = 21). (**D**) Recurrence versus primary (DEPs = 181). Each graph depicts normalized *z* scores for each detected master protein versus their corresponding adjusted *P* values. Dashed lines represent adjusted *P* value thresholds lower than 0.01 or 0.05 (*x* axis) or fold changes (FCs) greater than 2 or less than –2 (*y* axis). Significantly deregulated proteins shown in the upper right quadrant (orange circles) denote proteins overrepresented in the group under study, while proteins labeled by blue circles represent proteins overrepresented in the control group. Names of proteins functionally related to the complement system or S100A/ANXA protein families are included in **D**. Thresholds for significant adjusted *P* values were set according to the number of samples included in each comparison.

The comparison of the proteomic profiling of HGSOC cases versus controls revealed a differential PFD-sEV cargo for 485 proteins (Figure 6A and Supplemental Data 1), which represents more than 25% of the total number of identified proteins. Among them, 96 different proteins appeared significantly over-contained (52/96) or under-contained (44/96) when astringent thresholds were considered (*P* < 0.01 and *z* score > 3).

Subsequent analysis performed for other clinically relevant variables, such as the degree of response to platinum-based combinations, *BRCA* status, or the type of surgery that allowed PFD-sEV collection, showed a differential content between the groups under study of 22, 21, and 181 proteins, respectively (*P* < 0.05 and *z* score > 2; Figure 6, B–D, and Supplemental Data 1). The higher number of factors deregulated for the surgical procedure comparison is in fact in accordance with the ability of the correlation clustering analysis to distinguish among samples obtained at the diagnostic or primary intervention, after neoadjuvant chemotherapy, or at recurrences. It is noteworthy that the STRING database in silico analysis of proteins with differential content between patients with HGSOC undergoing recurrent surgeries versus primary or diagnostic procedures demonstrated an enrichment of factors related to the complement system and the S100A/ANXA protein families (Supplemental Figure 7A), which can also be observed in Figure 6D. As previously suggested, it also denotes that the ratio of cargo proteins responsible for the role of PFD-sEVs in cancer progression is higher than that of cargo proteins related to the molecular basis of HGSOC pathogenesis or chemoresistance.

### Validation studies support a pro-tumorigenic role of S100A4 and STX5 derived from PFD-sEVs in HGSOC cases.

**Figure 7:**
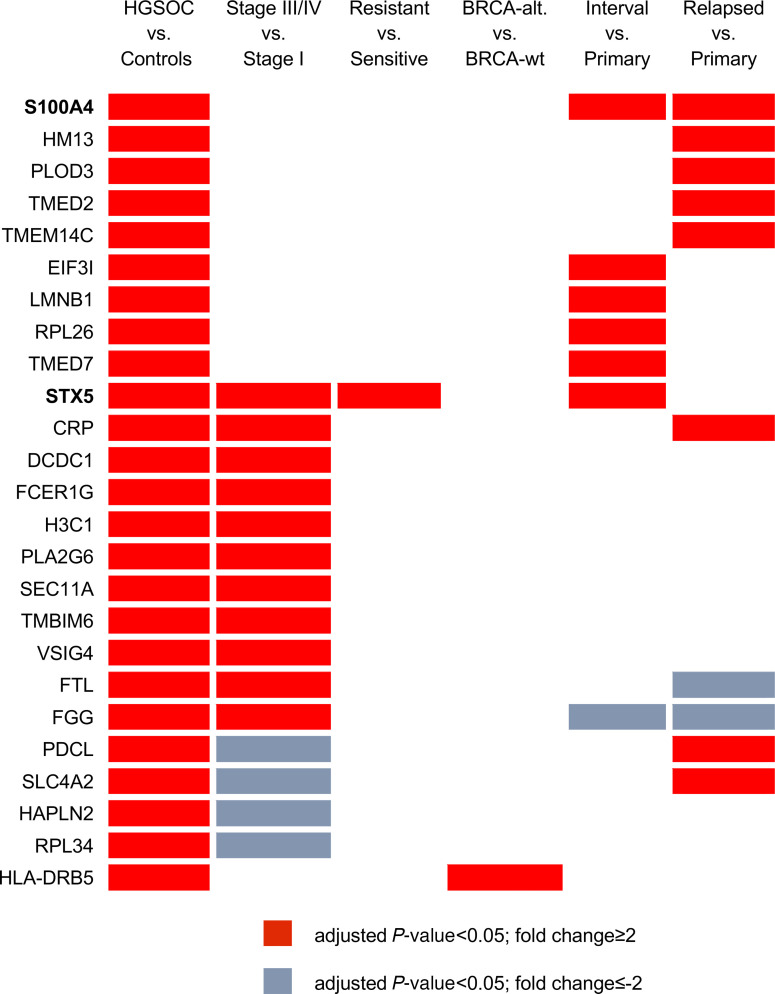
The 25 proteins that were differentially contained among different comparisons of interest. Red boxes represent proteins upregulated in PFD-EVs collected from the experimental group (fold change > 2), while cyan boxes indicate proteins upregulated in the control condition.

As presented in Figure 7, 25 of the identified proteins exhibited significant deregulation across various clinically relevant comparisons. Consequently, we established criteria for selecting targets of interest based on both their plausible biological role in tumor development and progression and expression ratios showing a consistent trend among the various comparisons under analysis. Considering these conditions, we directed our attention to 2 proteins among the 25 identified: S100A4 and STX5.

STX5 is actively involved in both autophagy events and the transport between cellular compartments (ER to Golgi). Our data set showed that *STX5* PFD-sEV–derived content was statistically higher both in patients with HGSOCs versus controls (fold change [FC] 4.55, adjusted *P* value 0.008) and in HGSOC platinum-resistant versus HGSOC-sensitive patients (FC 2.27, adjusted *P* value 0.015) (Supplemental Data 1). Such significant differences were also observed when individual *z* scores for STX5 content were considered (Supplemental Figure 7B and Supplemental Data 1). Regarding *S100A4* factor, extensive data support its role in promoting the development and acquisition of aggressive phenotypes in different solid carcinomas (24, 25). In agreement, our proteomic profiling showed that S100A4 PFD-sEV content was significantly higher in HGSOC cases versus controls (FC 2.00, adjusted *P* value < 0.001) and in relapses versus interval debulking or diagnostic specimens (FC 2.57, adjusted *P* value < 0.0001) (Supplemental Figure 7, B and C, and Supplemental Data 1). Additional data supporting the potential role of S100A4 in HGSOC pathogenesis were the progressive increase of its *z* scores throughout the clinical course of the disease (Supplemental Figure 7C) and its potential involvement with other functionally related proteins (ANXAs/S100A axis) (Supplemental Figure 7A).

**Figure 8:**
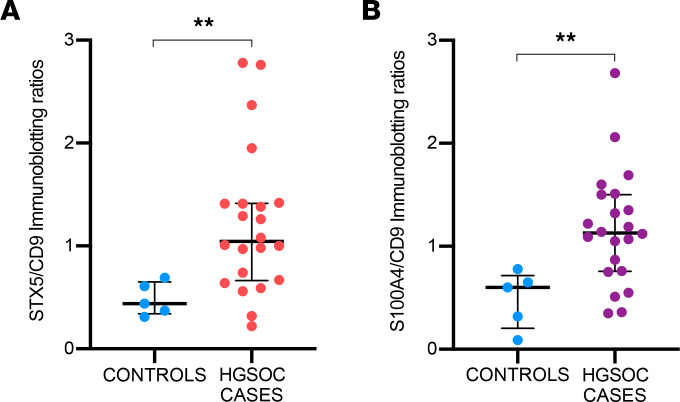
Graphs depicting results related to the validation of targets of interest. STX5/CD9 (**A**) or S100A4/CD9 (**B**) ratios obtained through immunoblotting in samples belonging to the validation cohort, including 5 controls and 22 patients with HGSOC. Western blot bands corresponding to the above-mentioned factors were quantified using ImageJ software, and the corresponding normalized ratios are depicted in this graph as individual dots. Data are shown as median and IQR from each independent sample/experiment. **P < 0.01, Mann-Whitney test.

Based on these assumptions, we performed a biological validation by Western blot on samples belonging to a new independent cohort of 22 patients with HGSOC and 5 controls not included in the mass spectrometry analysis. Notably, the PFD-sEV content of both STX5 and S100A4 targets was significantly higher in HGSOC samples compared with controls (STX5 *P* value = 0.0098; S100A4 *P* value = 0.008) (Figure 8, A and B, and Supplemental Figure 8A). In addition, subclassification of HGSOC cases according to the type of surgery confirmed a higher S100A4/CD9 ratio for any of the surgical procedures with respect to controls, detecting a consistent trend with relapse samples (*P* = 0.055) and showing robust differences when comparing with primary samples (*P* < 0.01) (Supplemental Figure 8B). Due to the narrow nature of our data set, there is a need to validate these observations in independent series; nevertheless, all these findings underline the potential role of PFD-sEV–contained proteins as factors involved in the development and progression of HGSOC.

### Functional enrichment analysis reveals molecular categories of interest: MTORC1 and complement/coagulation signaling pathways.

In silico tools (Enrichr package or gene set enrichment analysis [GSEA]) were used to identify biological processes and functional categories altered among the differentially PFD-EV–contained proteins or among a rank-ordered protein list, respectively. Enrichr analysis of the data obtained from the comparison between HGSOC and controls elucidated an enrichment of proteins associated with extracellular components such as vesicles, organelles, and, more specifically, PFD-sEVs (Supplemental Figure 9A and Supplemental Data 2), confirming the efficiency of our experimental approach and the robustness of our findings. Other relevant collections, such as the Molecular Signatures Database (MSigDB) Hallmarks and Reactome, revealed an overrepresentation of proteins involved in complement and coagulation cascades, oxidative phosphorylation, immune events, and the phosphatidylinositol-3-kinase (PI3K), mammalian target of rapamycin (mTOR), and RAS signaling pathways (Supplemental Figure 9, B and C, and Supplemental Data 2).

GSEA performed on the ranked lists based on proteomic quantifications for each comparison of interest (HGSOC cases vs. controls, resistant vs. sensitive, or recurrence vs. diagnostic) (Supplemental Figure 9D and Supplemental Data 2) revealed statistically significant enrichments in molecular categories similar to those defined by Enrichr. In this regard, we repeatedly observed associations with molecular classes related to the complement/coagulation pathway, cell-to-cell interaction (apical junctions) and motility (actin skeleton), estrogen-mediated signaling, and oxidative phosphorylation. Collectively, these findings suggest that while certain individual proteins may actively promote HGSOC progression, it is possible that specific functional processes or pathways promoted by protein sets contained in PFD-sEVs may also play a pro-tumorigenic role.

### Pathways over- and down-represented in sEV proteomic clusters from HGSOC cases could condition patients’ clinical outcomes.

PCA considering the 25 top proteins differentially up- or down-contained from the comparison of S-1 and S-2 clusters (Table 2) allowed us to clearly distinguish HGSOC samples included in each subset (Supplemental Figure 10A), suggesting that such a protein panel could represent a molecular signature set for the identification of patients likely to have prolonged overall survival. It is worth mentioning that the correlation between our prognostic signature and other well-validated signatures based on HGSOC tissue (26) demonstrated that, despite being grounded in different omics tools and based on distinct biological materials, there was a certain degree of correlation between both studies. In this regard, of the 126 genes belonging to the Yoshihara et al. signature (26), our study detected the expression of 8 (6.3%) of their corresponding proteins in all samples of our HGSOC cohort. Although only ANXA1 was included in our prognostic signature of 50 proteins, 6 of these 8 proteins (ANXA1, SERPINE1, APOL1, ALOX5AP, DSTN, and FCER1G; 75%) exhibited differential content in S-1 versus S-2 cluster comparison (*P <* 0.05), with the seventh protein (PGK1) showing values close to statistical significance (Supplemental Figure 10B).

Notably, the Enrichr analysis revealed enrichment of functional categories related to epithelial differentiation in proteins overrepresented in the S-1 cluster, where samples from salvage surgeries at relapse tended to accumulate (Supplemental Figure 10C). On the other hand, and in line with what Yoshihara et al. described (26), various categories related to immune processes were enriched among the overexpressed proteins in the S-2 cluster, where primary samples tended to cluster (Supplemental Figure 10D). These findings, which are in accordance with those observed in previous comparisons (HGSOC cases vs. controls and relapses vs. primary/diagnostic specimens, Reactome subset; Supplemental Figure 9, B and C), support a key role of immune processes in HGSOC tumorigenesis and are in line with prior communications associating the activity of the immune system with a better clinical outcome (27).

## Discussion

We present the results of a prospective observational study demonstrating how the characteristics of sEVs evolve dynamically during HGSOC progression and after exposure to platinum-based therapies. PFD-sEV proteomic profiling classified HGSOC carcinomas into 2 groups with different clinical characteristics and overall survival, and independent of other well-established prognostic factors such as *BRCA* status or platinum sensitivity. These proteomic profiles identified immune processes as key features potentially modulated by PFD-sEVs. Taken together, our data suggest a role for PFD-sEVs in the initiation, progression, and clinical outcome of HGSOC, although their potential as disease biomarkers and therapeutic targets requires further investigation.

sEVs have been extensively studied in OvCa in recent decades (28). Many authors have conducted mechanistic analysis of their role in oncological processes such as cancer initiation, tumor dissemination, sensitivity to chemotherapy, and modulation of the tumor microenvironment (29, 30). However, these in vitro studies relied mostly on noncoding RNA and have had little translation into the clinic. Another relevant line of investigation focused on the assessment of the potential of serum sEVs as early diagnostic tools. In this regard, several microRNAs and microRNA signatures (14, 15, 31) and, to a lesser extent, sEV proteins (16) have been detected in OvCa while being absent in healthy controls. However, as expected in a tumor with limited hematogenous spread, the proportion of tumoral sEVs is minimal, and the correlation with clinical outcome was uncertain in most cases.

In our study, we collected peritoneal washings and malignant ascites from patients with OvCa undergoing any oncological surgery. This approach aimed to yield maximal amounts of tumor sEVs, enabling us to conduct high-throughput proteomic analysis of OvCa-derived vesicles. Additionally, we defined patterns of sEVs at different time points and evaluated their evolution in serial samples. The preference for studying proteins over noncoding RNAs was based on their role as a true paracrine system linking tumor cells and their environment (32). Thus, our study evaluates the potential association between clinical features and outcome and tumor sEVs and their proteomic profiles in OvCa.

The characterization of the PFD-sEV protein content within our OvCa cohort enabled us to confirm its malignant origin. Specifically, PAX8 expression, a marker of tumor cells (serous, endometrioid, and clear cell OvCa) derived from the fallopian tube secretory cell and less frequently expressed in benign pathologies (33, 34), was detected in all OvCa cases, but solely in 1 control.

Additionally, our study corroborates the results of previous proteomic studies in which differentially expressed proteins were determined in paired specimens from patients with OvCa (tumor vs. normal tissue) or comparing OvCa and control samples (urine and serum) (23). In this regard, we confirmed a significant overexpression of 7 markers (PEBP1, LGALS3, S100A8, FTL, PSMA6, COL3A1, and AFM) when PFD-sEV cargo from HGSOC cases versus controls was compared. Minor differences observed for the remaining biomarkers could be attributable, not only to the different origin of the samples, but also to the contribution of the tumor microenvironment to the proteomic profile of OvCa-derived PFD-sEVs. In this sense, Lai et al. recently proposed an OvCa-diagnostic signature based on the isolation of sEV-derived serum markers (FGG, APOA4, and MUC16 [also known as CA125]) (16). Although we confirmed the diagnostic role of FGG and APOA4 in the PFD-sEV fraction, this was not the case for MUC16. Our results align with most prior clinical studies that have extensively tried to validate CA125 as a marker of OvCa. Unfortunately, despite the large numbers of patients included in these studies, the specificity of this approach remains limited and must be interpreted alongside additional diagnostic procedures (5, 35).

Our approach has also demonstrated that tumor stage, clinical progression, and the degree of exposure to chemotherapy strongly condition both the number and protein content of PFD-sEVs in HGSOC, not only quantitatively but also qualitatively. In this sense, a significant association between tumor stage or progression and the number of sEVs has been previously described in different types of solid tumors, such as melanoma and lung (15, 36). In the context of epithelial OvCa, it has been shown that different tumor-associated cell types (cancer cells, cancer-associated fibroblasts, and immune component, among others) determine a high degree of heterogeneity in vesicle composition at the local and distal tumor environment (32), consequently promoting several pro-tumorigenic processes (37, 38). Such tumor-associated sEV heterogeneity would be consistent with the fact that our correlation clustering analyses based on PFD-sEV profile classified HGSOC according to disease status, being able to discriminate paired samples depending on the studied time point. Concerning the benign pathologies within the control cohort, the various histological entities were distributed evenly across 2 clusters when compared with HGSOC cases. In this regard, it can be argued that the collected sEVs originated from nonpathological cells or abdominal structures, rather than from gynecological lesions. This observation is supported by the fact that PAX8 protein, a marker of gynecological pathologies, was isolated exclusively in one of the nononcological controls.

Given that alterations in *BRCA* or HRD status do not substantially alter the differential profile of PFD-sEVs, it is worth noting that both tumor stage and progression and chemotherapy represent major forces of vesicle variability in HGSOC. Of particular interest are the results obtained from serial samples in which patients underwent 2 or more separate surgical interventions over time. Except for a single case that exhibited an opposite behavior, the number of vesicles increased while the protein concentration per vesicle decreased after neoadjuvant treatment. Although this result is challenging to interpret, it is possible that the tumor cytolysis induced by chemotherapy led to this dual modification of sEV-related features. This hypothesis is based on the premise that the physical factors (e.g., permeable vasculature, mechanical stress) and environmental conditions (acidic or hypoxic environments and cisplatin-based therapies, among others) in which the tumor grows alter vesicle cargo and secretion ratios. (32, 39, 40). However, considering that the patient displaying an opposite trend showed no differences in clinical outcome, the significance of these changes should be explored in additional cohorts.

Accordingly, the study of these dynamic changes occurring in the content of PFD-sEVs may contribute to the identification of prognostic signatures, as demonstrated by the significant correlation of their proteomic profile with OS in our cohort of patients with HGSOC. Although based on different molecular approaches (transcriptomic vs. proteomic) and different biological samples (tumor tissue vs. PFD-sEVs), the correlation observed between the prognostic signature described by Yoshihara et al. (26) and our proteomic data corroborates the prognostic potential of characterizing the content of PFD-sEVs in HGSOC. Of particular interest is the enrichment in categories related to the immune system, whose prognostic role was also addressed in the study by Yoshihara et al. Consistent with these findings, several OvCa studies have demonstrated how ascites-derived sEVs induce in vitro inactivation of CD3 and CD8 lymphocytes (41, 42) and activation of M2 macrophages (43) leading to disease progression. Globally our results not only confirm the role of these tumor-related sEVs in the modulation of the immune microenvironment, but also demonstrate their association with HGSOC disease outcome. However, this potential should be further determined in future studies.

Detailed analysis of the hallmark traits enriched in our proteomic data comparisons (HGSOC vs. controls, relapses vs. diagnostic/primary samples) revealed a recurrent association with categories such as the complement/coagulation cascade and immune responses, the PI3K/AKT/mTORC pathway, different metabolic processes, and hypoxia. The increased expression of complement factors (C4A, C4B, C5, and C1R/S, among others) in PFD-sEVs between diagnostic samples and controls or recurrence samples could be related to previous findings suggesting that extracellular activation of this cascade by TAMs represents an innate mechanism of immunosuppression that maintains a chronic inflammatory status promoting tumor progression (44, 45). Furthermore, it is worth noting the close relationship between peritoneal inflammation and the onset and progression of epithelial OvCa (46). On the other hand, oxidative phosphorylation and fatty acid processing were among the metabolic changes deregulated in our study. The involvement of these processes is consistent with the metabolic reprogramming that ovarian tumors may undergo, allowing them to prioritize oxidative phosphorylation over glycolysis to fuel tumor cells under hypoxic conditions (47). Notably, OvCa dependence on oxidative phosphorylation has been associated not only with increased survival (48) and proliferation of cancer-initiating stem cells, but also with increased chemosensitivity of tumor populations with high oxidative phosphorylation ratios (48), therefore representing a promising therapeutic strategy. All these premises indicate that PFD-sEV content is a faithful reflection of tumor progression and its intrinsic cell heterogeneity.

Considering previously published data regarding our set of deregulated proteins, we proceeded to the biological validation of proteins of interest (STX5 and S100A4) using immunoblotting as an alternative technique for protein cargo evaluation. STX5 is a member of the SNARE protein family that functions as an integral membrane protein playing a crucial role in autocrine and paracrine signaling through exocytosis and vesicle fusion–related events. Multiple oncogenic properties have been widely linked to the expression of SNARE proteins (49). Moreover, STX5 has been recently described to modulate PI3K/mTOR pathway activation, subsequently restraining cell adhesion and thus favoring metastasis in hepatocellular carcinoma (50). Although STX5-mediated pro-tumorigenic mechanisms in HGSOC remain to be elucidated, our results demonstrate that expression of STX5 in PFD-sEVs is associated with tumor progression and, to a lesser extent, with the degree of response to chemotherapy, which would be in line with analogous functions observed in other members of its protein family (49). Conversely, further literature links the overexpression of both S100A4 and other members of its family to the development of metastasis through both intra- and extracellular functions. Several tumor components (cancer cells, cancer-associated fibroblasts, or immune cells) are capable of secreting S100A4, highlighting that its pro-tumorigenicity may be mediated by its inclusion in sEV cargo (51, 52). Alluding to its involvement in OvCa development, several studies have described its role in metastasis induction and chemoresistance (25, 53), and its potential use as a liquid prognostic marker for these neoplasms (54). In line with these assumptions, our results demonstrate that the oncogenic role of S100A4 in HGSOC is mediated, to some degree, by its secretion via PFD-sEVs and that it could serve as a biomarker of prognostic interest for HGSOC.

Our prospective study, which included massive proteomics on PFD-sEVs from patients with OvCa, has allowed us to perform a longitudinal screening of the clinical course of the disease, demonstrating that the study of PFD-sEV content can be useful in the identification of prognostic signatures. Restricting this analysis to the peritoneal cavity, we have further defined pro-tumorigenic proteins and molecular pathways potentially involved in the intercellular communication of the tumor microenvironment, in line with the expected tumor heterogeneity of sEV content. Further studies in larger cohorts are required to validate the findings of this study.

## Methods

### Sex as a biological variable.

Our study exclusively focused on female samples, as the disease under investigation (OvCa) is only relevant in females.

### Clinical and molecular parameters for the categorization of patients with OvCa.

Given that the OvCa patient samples were obtained from different types of surgeries, the nomenclature for these procedures is detailed as follows: “primary” indicates primary surgery performed as the initial therapeutic maneuver or diagnostic laparoscopy; “interval” indicates interval surgery after neoadjuvant chemotherapy; “relapse” indicates salvage surgery of recurrent disease. The surgical outcome terminologies are defined as follows: R0 is assigned when there is no residual macroscopic disease after surgery; R1 is attributed for any residual disease after surgery (regardless of the size). “NA” (“not applicable”) is used for those patients who underwent diagnostic laparoscopy procedures, in which only a fragment of the tumor was excised for diagnosis purposes. Patient samples were classified based on patient sensitivity to the next platinum-based chemotherapy administered after the collection of the sample; platinum-sensitive patients were defined as those with a platinum-free interval (PFI), defined as the time from last chemotherapy to tumor relapse, greater than 6 months, while platinum-resistant patients were considered those with a PFI less than 6 months. *BRCA* status in patients was defined as follows: BRCA wild type (WT), referring to the absence of pathogenic alterations in *BRCA1/2*; and *BRCA* altered (ALT), indicating the existence of *BRCA* locus harboring a pathogenic alteration. Regarding the status of the homologous recombination (HR) pathway, HRD status reflects homologous recombination–deficient tumors, while HRP denotes tumors with homologous recombination–proficient features.

### Sample collection, PFD-sEV isolation, and NTA profiling.

Immediately after the collection of peritoneal lavages from surgeries, centrifugation was performed at 1,500*g* for 10 minutes to remove tissue debris, and the supernatant was stored at –80°C. Isolation and purification of sEVs from peritoneal fluid were systematically carried out using previously described protocols (21). Briefly, the samples were centrifuged at 3,000*g* for 20 minutes, followed by an additional centrifugation of the supernatant at 12,000*g* for 20 minutes in the Beckman Optima L-90K ultracentrifuge (Beckman Coulter) using the Type 55.2 rotor. sEVs were concentrated by centrifugation at 100,000*g* for 70 minutes. The resulting pellet was washed in 5 mL PBS and collected through a second ultracentrifugation at 100,000*g* for 70 minutes using the SW55 rotor. The resulting pellet was resuspended in 100 μL of PBS, and the protein content was measured by bicinchoninic acid (BCA) assay (Pierce). Particle number was measured from an aliquot of 1–2 μL of sEVs diluted in 1 mL of PBS using NTA (NanoSight, Malvern Panalytical Ltd.) equipped with a violet laser (405 nm). For each sample, we recorded three 1-minute video clips.

### Transmission electron microscopy.

For negative staining, 5 μL of the purified fractions resuspended in 2% PFA at a concentration of 1 × 10^11^ particles/mL were each deposited on a parafilm layer. A grid coated with formvar/charcoal was placed over each drop, and the sEVs were allowed to adsorb for 20 minutes. Then they were washed over 5 drops of 100 μL of PBS for 1 minute each, and subsequently each grid was placed over a drop of 50 μL of 1% glutaraldehyde in PBS and fixed for 5 minutes. After this, the grids were washed over 8 drops of 100 μL of distilled water for 2 minutes each and placed on a drop of 50 μL of uranyl-oxalate, contrasted for 5 minutes, and left to dry. Finally, the grids were placed on a drop of 50 μL of methylcellulose–uranyl acetate for 10 minutes on ice and allowed to dry at room temperature. Visualization of the grids was performed on a JEOL JEM1010 transmission electron microscope (100 kV). Images were recorded with a Gatan Orius 200 SC digital camera.

### Immunoblotting.

sEV protein content was measured by BCA, and 10 μg aliquots were used for Western blot analysis. sEVs were lysed in Laemmli buffer at 96°C for 5 minutes to denature proteins, and protein extracts were resolved by SDS-PAGE. Antibodies were tested against albumin (sc-271605, Santa Cruz Biotechnology), apolipoprotein B (ab139401, Abcam), TSG101 (ab125011, Abcam), CD9 (ab58989, Abcam), and ALIX (ab88743, Abcam). Antibodies were chosen according to MISEV 2023 guidelines (21). Immunostaining against PAX8 (PA 0300, Biopat), a well-known ovarian carcinoma marker, was also incorporated in the Western blot characterization panel. The biological validation used antibodies against STX5 (HPA001358, Sigma-Aldrich) and S100A4 (ab93283, Abcam). Clarity ECL Western Blotting Substrate (Bio-Rad) was used to visualize the bands and developed on the ChemiDoc imaging system (Bio-Rad). The intensity of the immunoreactive bands was quantified by densitometry using ImageJ (NIH).

### Proteomic analysis of PFD-sEVs by LC-MS/MS through differential analysis with TMT18-plex isobaric tagging.

For the proteomic analysis, LC-MS/MS was performed using an Ultimate 3000 Nano HPLC liquid nanochromatography system (Thermo Fisher Scientific) coupled to an Orbitrap Exploris 240 mass spectrometer (Thermo Fisher Scientific). Once the sEVs were isolated, they were concentrated on Nanosep columns (Omega; 10 kDa) with a starting volume greater than 50 μL. They were then lysed with 4% SDS, followed by reduction and alkylation of cysteines using TCEP and MMTS as reducing and alkylating reagent, respectively. A tryptic digestion was then performed on Protifi’s commercial S-Trap columns. Peptide quantification (QuBit, Invitrogen) was then carried out. Finally, isobaric tagging (TMT18-plex) was performed with 20 μg of peptide mixture per condition. In this strategy, each sample is labeled with a different isotopic version of the reagent and combined into a single sample for MS analysis.

The generated tryptic peptides were separated by liquid chromatography and desolvated and ionized before entering the Exploris 240 high-resolution mass spectrometer (with Orbitrap analyzer), where they were first detected as *m*/*z* ions in the range 375–1,200 *m*/*z*, and then the 20 most intense ones were taken, from which MS/MS fragmentation spectra were obtained for identification. A long length gradient (120 minutes) was applied on a C18 column of 75 μm ID and 50 cm length. The different isotopic versions of the reagent were chemically identical, so that the same isotopically labeled peptide would generate the same precursor ion in the MS1 spectrum, differing only in the *m*/*z* values of the reporter or control ions characteristic for each of them. The relationship between the intensities of these control ions in the fragmentation spectra provided quantitative information at the peptide level, which in turn was related to differential expression levels at the protein level between samples.

### LC-MS/MS analysis and data extraction.

Proteins were identified in the raw files using the SEQUEST HT algorithm integrated into Proteome Discoverer 2.5 (Thermo Finnigan, Thermo Fisher Scientific) (55). MS/MS scans were matched against the UniProtKB human proteome database (2022_06 release) concatenated with the reverse decoy database obtained from the DecoyPYrat program (James Christopher Wright, Wellcome Trust Sanger Institute).

The parameters selected for the database searching were as follows: trypsin digestion with 2 maximum missed cleavages allowed, precursor mass tolerance of 800 ppm (2 Da), and a fragment mass tolerance of 0.02 ppm (0.02 Da) (56). The N-terminal and lysine TMT18-plex modifications and the cysteine carbamidomethylation were chosen as fixed modifications, whereas the N-terminal acetylation, the methionine oxidation, and Gln→pyro-Glu (N-terminal Q) were chosen as variable modifications. The false discovery rate (FDR) was calculated using the refined method based on the results obtained by database searching. Quantitative information was extracted from the intensity of the TMT18-plex reporter ions in MS/MS spectra.

### Bioinformatic analysis.

For comparative analysis of protein abundance changes, we used the weighted spectrum, peptide, and protein (WSPP) statistical model in the SanXoT software package (57). The model provides a detailed description of the behavior of technical variance, and by analyzing it independently at the spectrum, peptide, and protein levels, the model is able to capture separately the specific error sources of each SIL and MS method, demonstrating that error distributions are accurately modeled in all cases at the 3 levels. In addition, this model provides a standardized variable, Zq, defined as the mean-corrected log_2_-ratio expressed in units of standard deviation at the protein level. ConsensusClusterPlus R package (58) was used to perform hierarchical consensus clustering on protein expression data of control and HGSOC samples. A maximum of 6 clusters were fixed, and the algorithm was trained over 10,000 repetitions using 80% of the samples. Pearson distance was used with complete hierarchical clustering. Results show a stable structure of 5 clusters (Supplemental Figure 4D), 2 of them belonging to HGSOC cases.

### Systems biology.

For the analysis of coordinated protein changes, we used both GSEA (59) and Enrichr (https://maayanlab.cloud/Enrichr/) in silico tools. GSEA calculates a normalized enrichment score that measures the degree of enrichment and uses a permutation test to calculate a *P* value that determines the statistical significance of the enrichment. The FDR (60) approach is used to control for multiple testing and reduce the likelihood of false positives. GSEA contains multiple molecular signature databases, including Gene Ontology, KEGG, and Hallmarks. With respect to Enrichr, those differentially expressed proteins (adjusted *P* value < 0.05) for each comparison of interest were included in the analyses, which included relevant functional categories (Hallmarks, Reactome, and Transcription Factor [TF]-Gene Co-Occurrence).

### Statistics.

Prism (version 8.1.1, GraphPad Software) was used to perform all statistical analyses. Assessment of all particle and PFD-sEV concentrations, quantitative analysis of protein content per particle per 10^9^ in PFD-sEVs, measurement of normalized ratios (PAX8/CD9, STX5/CD9, and S100A4/CD9) through immunoblotting in both control and OvCa samples, and comparisons of proteomic cargo between control and OvCa cases were performed using the nonparametric Mann-Whitney test. For multiple comparisons, *P* values were adjusted using the Benjamini-Hochberg method. For analyses involving more than 2 groups, the Kruskal-Wallis test was used, followed by Dunn’s post hoc multiple comparisons with Bonferroni’s *P* value adjustment. Results are expressed by median and IQR. Analyses of PFD-sEV concentration and protein content per particle per 10^9^ in PFD-sEVs among the paired samples were conducted using the parametric paired 2-tailed *t* test. Kaplan-Meier estimator and Cox method were performed for survival analyses. Different survival curves were compared using the log-rank test. In addition, the limma package was used to determine differentially expressed proteins between OvCa-specific clinical variables and controls. *P* values less than 0.05 were considered statistically significant.

### Study approval.

The study protocol (21.01.1289E1-GHM) for sample collection was approved by Ethics and Drug Research Committee, (HM Hospitals, Madrid, Spain) and patients provided written consent before being included in the study.

### Data availability.

Every value of the data points depicted in the graphs is accurately reflected in the Supporting Data Values file. Additionally, proteomic and differentially contained protein data are available in the Supplemental Data 1 file. For the Enrichr and GSEA analyses (Supplemental Figure 9, C and D, and Supplemental Figure 10, C and D), data values are provided in the Supplemental Data 2 file.

## Author contributions

MQ, SRL, and JGD designed the research studies. All experiments and data acquisition were done by MQ, AB, MYF, PN, ABS, and SRL. Histological assessments were performed by MP. Human clinical sample collection and patient assessments were performed by AB, ESF, JFRM, CM, ILC, and JGD. Proteomic profiling data were analyzed by MQ, SRL, RM, and IFM. HP and EI reviewed data, provided experimental advice, and contributed to manuscript editing and revision. The manuscript was written by MQ, TPG, SRL, and JGD. The project was conceived and supervised by SRL and JGD. All authors reviewed the manuscript and provided feedback on it.

## Supplementary Material

Supplemental data

Supplemental data set 1

Supplemental data set 2

Unedited blot and gel images

Supporting data values

## Figures and Tables

**Table 2:**
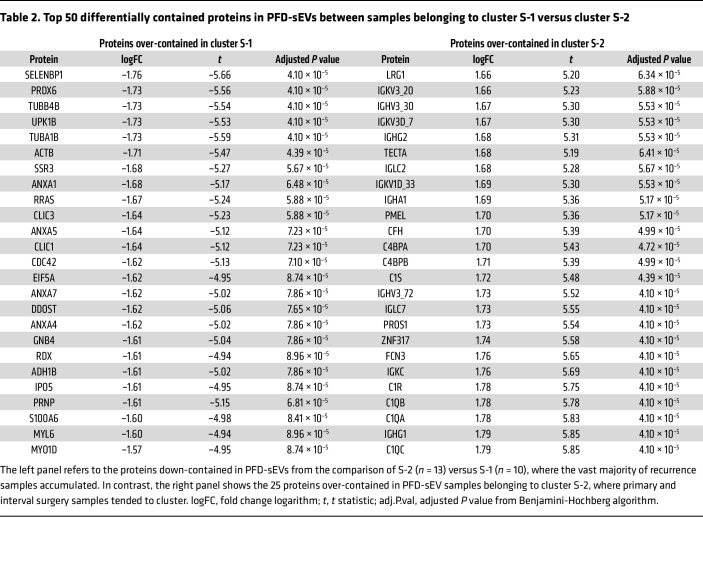
Top 50 differentially contained proteins in PFD-sEVs between samples belonging to cluster S-1 versus cluster S-2

**Table 1:**
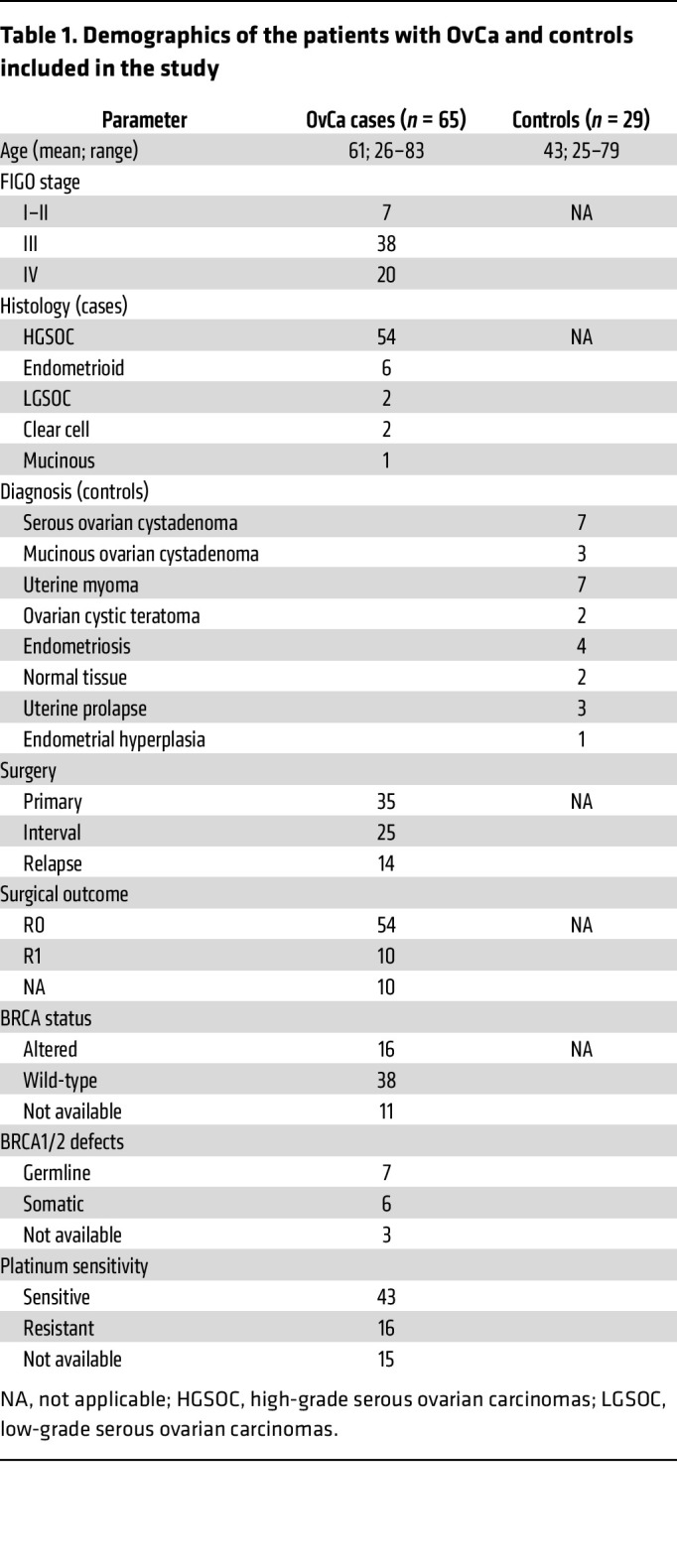
Demographics of the patients with OvCa and controls included in the study

## Version 1 04/02/2024

In-Press Preview

## Version 2 05/15/2024

Electronic publication
