# Supplementary material for: Proteomic profiles of peritoneal fluid-derived small extracellular vesicles correlate with patient outcome in ovarian cancer
Source: J Clin Invest. 2024 Apr 2;134(10):e176161. doi: 10.1172/JCI176161 (PMC11093605; doi:10.1172/JCI176161)
Supplement: Supplemental data [file jci-134-176161-s068.pdf]

SUPPLEMENTAL FILES

Supplemental Figure 1. (A) PDF-sEVs levels according to the OvCa histology. Additional information is provided regarding disease stage. The samples analysed included controls (CT n=29), high grade serous (HGS, n=63) and non-high grade serous (NON-HGS, n=11) composed of endometrioid (n=6), low grade serous (n=2), clear cell (n=2) and mucinous subtypes (n=1). **(B)** PDF-sEVs concentration in HGSOC classified according to the absence (R0) or presence (R1) of residual disease after cytoreductive surgery (R0=43 vs. R1=10). **(C)** PDF-sEVs concentration in HGSOC classified according to the sample source (peritoneal washings=44 vs. ascitic fluid=19). Data are represented by Median and interquartile range (IQR) from each independent samples/experiments. *P*-values of significant findings (**, p-value<0.01, Kruskal-Wallis test with Dunn's multiple comparison test and Bonferroni adjusted p-values [A]).

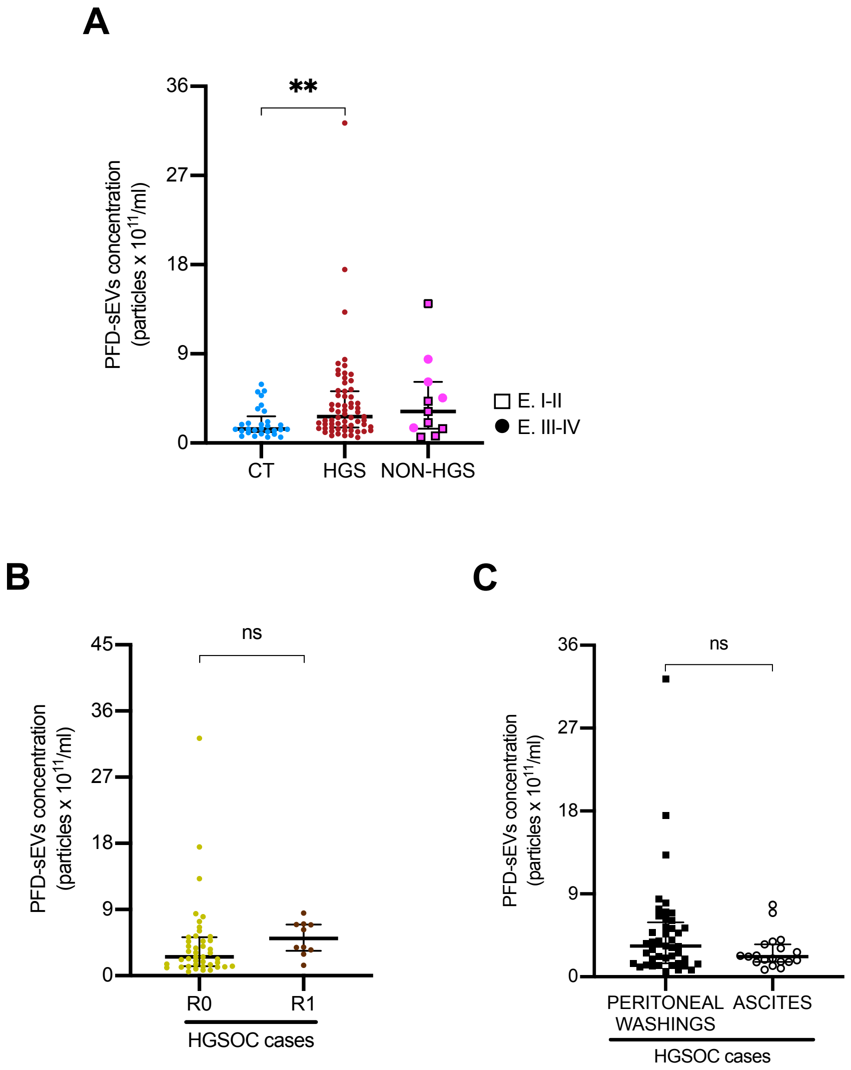

Supplemental figure 2. (A) Ratio of protein per particle in controls (CT n=29), HGSOC (n=63) and Non-HGSOC (n=11, composed of endometrioid [n=6], low grade serous [n=2], clear cell [n=2] and mucinous subtypes [n=1]) case samples. **(B)** Pearson correlation analysis of particle concentration and PCP in HGSOC. Each surgical procedure is shown in different colours (primary surgeries (n=22) in orange, interval surgeries (n=21) in green and relapses (n=12) in black). **(C)** Protein content per particle (PCP) in HGSOC patients classified according to the absence (R0) or presence (R1) of residual disease after cytoreductive surgery (R0=43 vs. R1=10). **(D)** PCP in HGSOC patients classified the sample source (peritoneal washings=44 vs. ascitic fluid=19). **(E)** Representative immunoblotting evaluating PAX8 expression in a selected panel of PDF-sEVs samples. Data are represented by Median and IQR from each independent samples/experiments. *P*-values of significant findings (*, p-value<0.05 and **, p-value<0.01, Kruskal-Wallis test with Dunn's multiple comparison test and Bonferroni adjusted p-values [A] or Mann-Whitney test [D]).

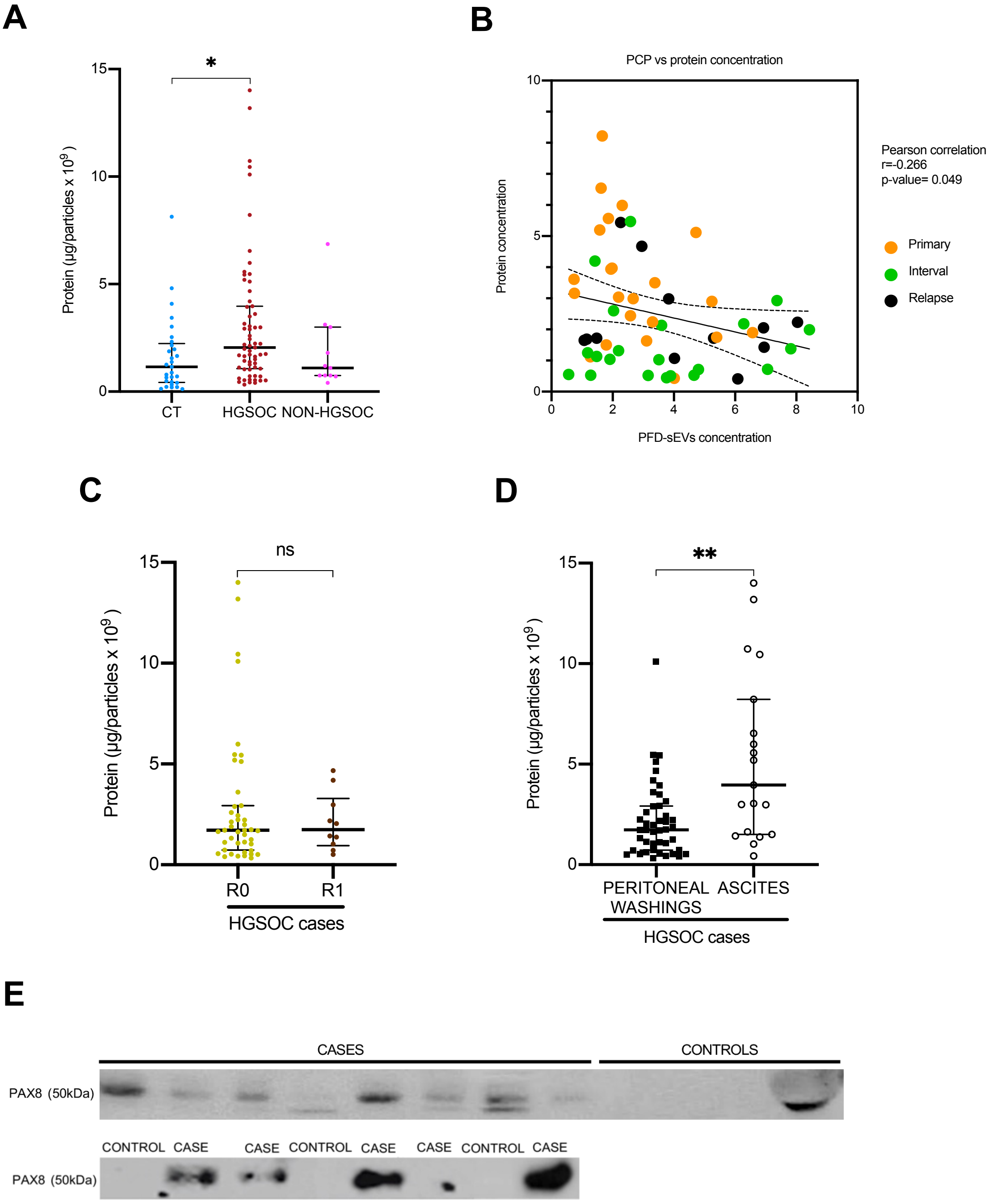

Supplemental figure 3. **(A)** Table depicting previous data from Toss et al. (23) related to the expression of prognostic markers determined by proteomic profiling in different specimens of OvCa patients and their correlation with our PDF-sEVs proteomic data. **(B)** Graph depicting the proteomic normalized quantification data for nine proteins with similar expression ratios compared to previously published data, which considered such factors as OvCa protein biomarkers. Comparison is established between controls (Ct, n=10) and HGSOC cases (Cases, n=23). Dark vertical lines separate markers previously described by 4 independent studies, which were referred in Toss et al. (23). Data are represented by Median and IQR from each independent samples/experiments.

A

Authors (PMID)	Samples collected for previous study	Sample set	Significance of the findings	Identified biomarkers	Alias name	Regulation in previous data	Our study (OvCa PDF-sEVs)
Cortesi et al. (21728179)	OvCa vs. normal tissues	Patients with endometrioid (n=3) or serous (n=3) ovarian carcinoma subtypes	Alterations in proteins that control cell-cycle progression and apoptosis may be used as a diagnostic and/or prognostic biomarker.	Phosphatidylethanolamine-binding protein 1	PEBP1	Downregulated	p-value<0.001
				Galectin-3	LGALS3	Downregulated	p-value<0.05
				Annexin-5	ANXA5	Upregulated	same expression trend
				Protein S100-A8-calgranulin A	S100A8	Upregulated	p-value<0.05
				Retinol binding protein	RBP1	Downregulated	same expression trend
An et al. (16674097)	OvCa vs. normal tissues	4 serous, 5 mucinous, and 3 endometrioid fresh-frozen ovarian tumors	Proteomic profiles differ according to histological subtype and tumor aggressiveness.	Ferritin light chain	FTL	Upregulated	p-value<0.001
				Proteasome alpha-6	PSMA6	Upregulated	p-value<0.01
				Annexin-1	ANXA1	Upregulated	same expression trend
Petri et al. (19023702)	Urine samples	156 benign tumors, 13 borderline tumors and 40 malignant epithelial ovarian cancer	Urine proteomic profiling represent a feasible strategy to identify novel diagnostic markers in ovarian epithelial carcinomas.	Fibrinogen alpha fragment	FGA	Upregulated	same expression trend
				Fibrinogen beta NT fragment	FGB	Upregulated	same expression trend
				Collagen alpha 1 (III) fragment	COL3A1	Upregulated	p-value<0.01

Supplementary figure 3A (continued)

Authors (PMID)	Samples collected for previous study	Sample set	Significance of the findings	Identified biomarkers	Alias name	Regulation in previous data	Our study (OvCa PDF-sEVs)
Jackson et al. (18094419)	Serum samples	*Discovery set:* 15 patients with ovarian cancer or benign ovarian cysts vs. 5 healthy controls. *Validation set*: 303 individuals	The evaluation of serum glycoproteins identified this target as a complementary marker to the use of Ca125 in the longitudinal monitoring of patients with ovarian cancer.	Vitamin E-binding plasma protein, Afamin	AFM	Downregulated	p-value<0.001
Li et al. (19056166)	Ovarian epithelial serous cystadenocarcinoma vs. normal tissues	16 normal epithelial tissues, 16 benign tumors, 21 serous cystadenocarcinoma, 16 mucinous cystadenomas and 2 other epithelial ovarian carcinomas.	Proteomic screening represents a useful strategy for the molecular characterization of the progression and treatment of malignant ovarian neoplasms.	Heat shock protein 60	HSPD1	Upregulated	same expression trend
				Prohibitin	PHB1	Upregulated	same expression trend

B

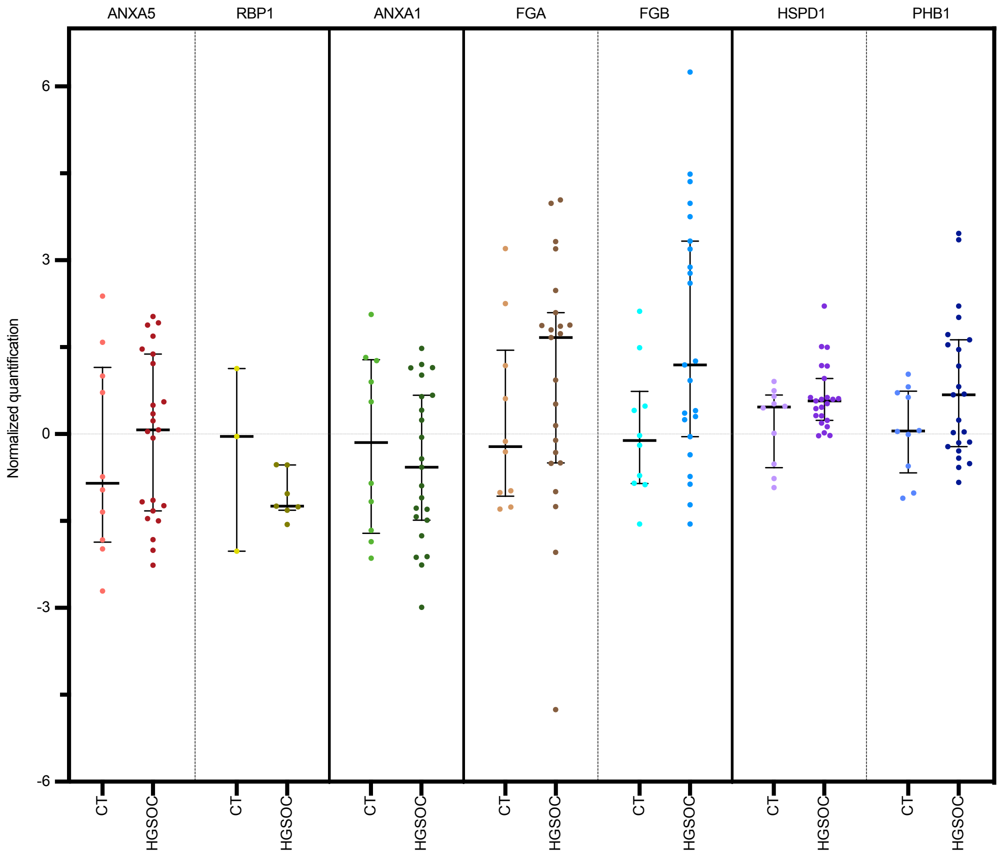
ANXA5
RBP1
ANXA1
FGA
FGB
HSPD1
PHB1
Normalized quantification
6
3
0
-3
-6
CT
HGSOC

Supplemental 4A-B. Correlation clustering map depicting the unsupervised analysis for primary (n=10) (Figure 4A) or interval (n=7) HGSOC samples (Figure 4B) combined with relapsed HGSOC specimens (n=6). The color bar on the left indicates the degree of correlation between two samples under study, with a value of 1 (dark red) indicating an identical sample in terms of protein cargo and -1 (dark blue) indicating potential samples with completely opposite profiles. On the right side of each panel, the main clusters and OvCa procedures (primary (P), interval (I) or relapse surgeries (R)) are shown. Relapsed samples preferentially accumulated in clusters R from each of the panels (5 out of 6), while primary samples tend to co-occur in the cluster P (8 out of 10) and neoadjuvant specimens in the cluster I (5 out of 7).

A

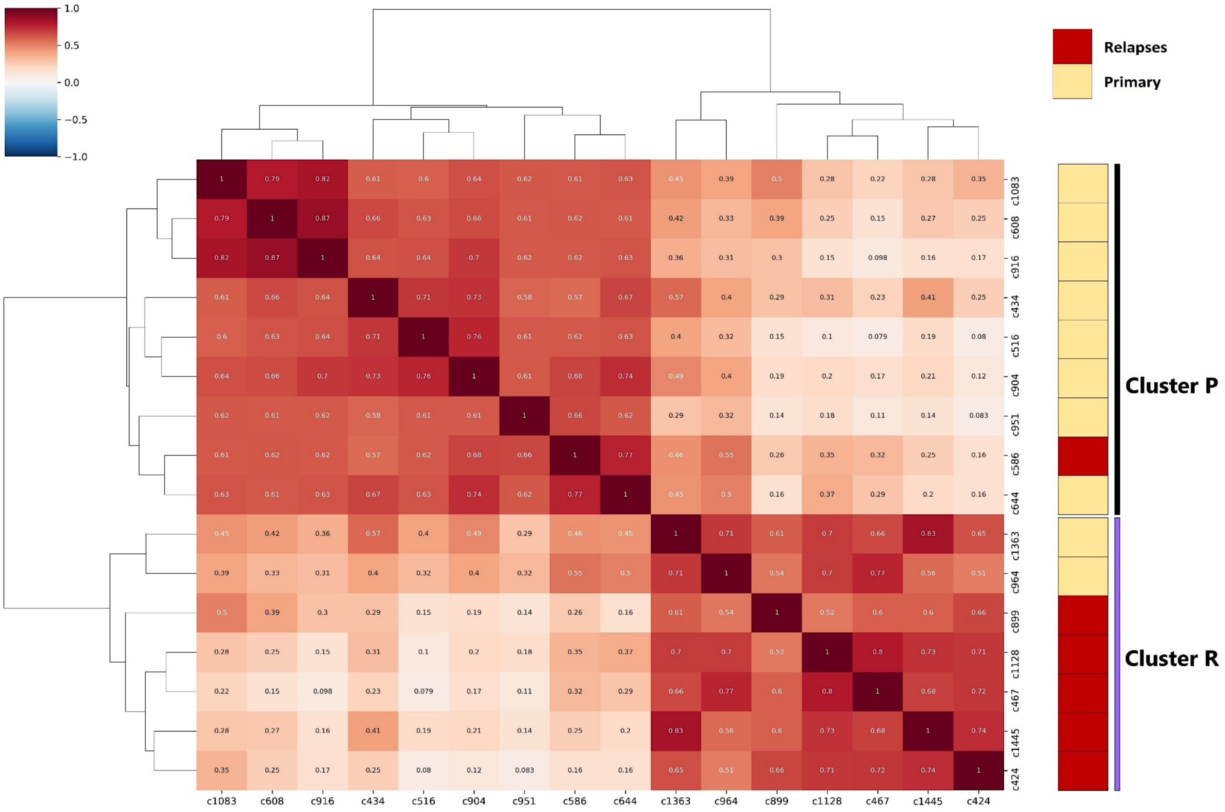

B

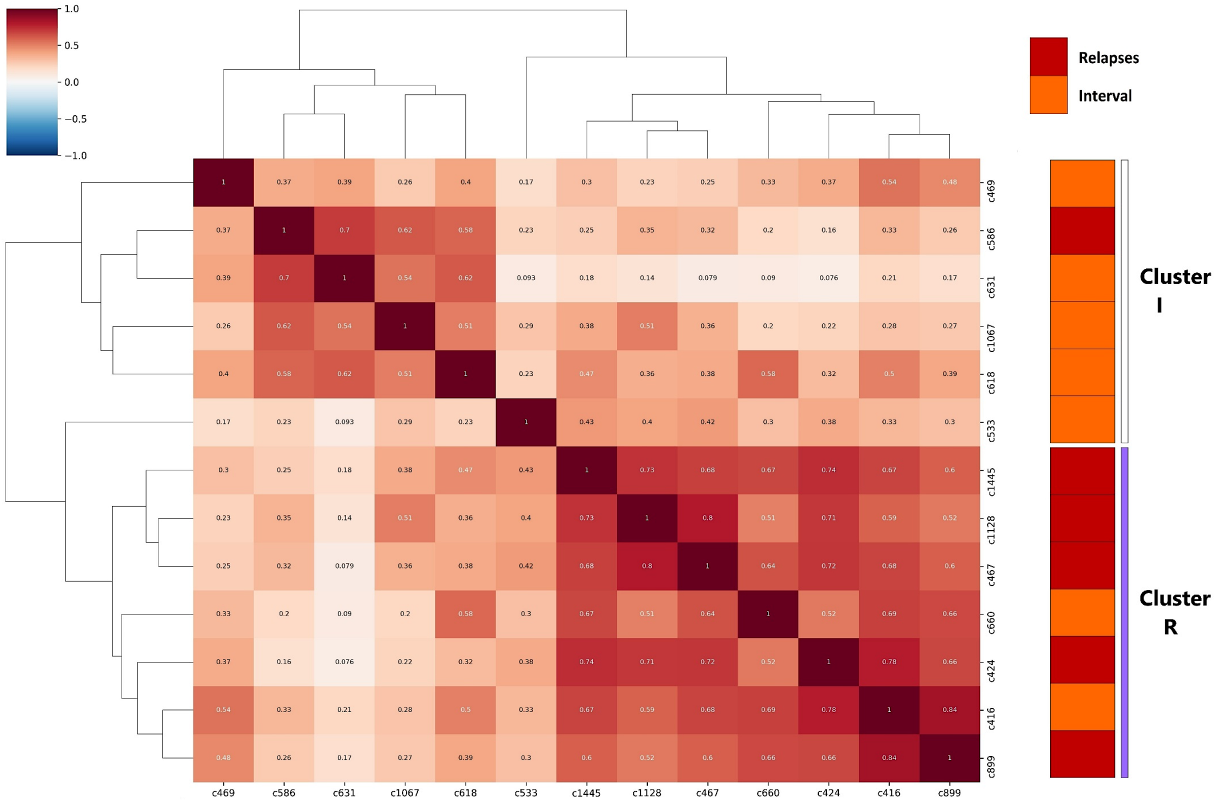
1.0
0.5
0.0
−0.5
−1.0
Relapses
Interval
Cluster I
Cluster R
c469
c586
c631
c1067
c618
c533
c1445
c1128
c467
c660
c424
c416
c899

Supplemental 4C. PCA analysis based on proteomic data obtained by comparing samples from control patients with all ovarian carcinomas under study (upper figure) or with HGSOC patients (lower figure). Both figures indicate the main sample groupings, which correlate with the subclusters, or clusters specifically described in main Figures 4A or 4B

OVCA & control samples

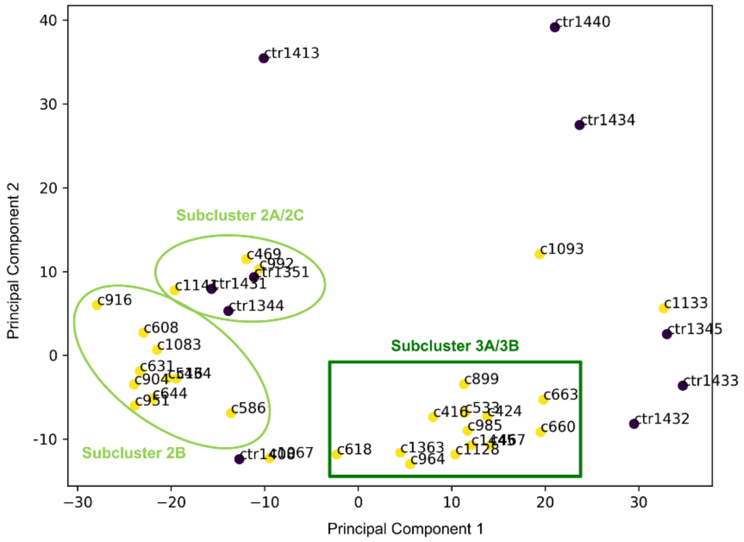

HGSOC & control samples

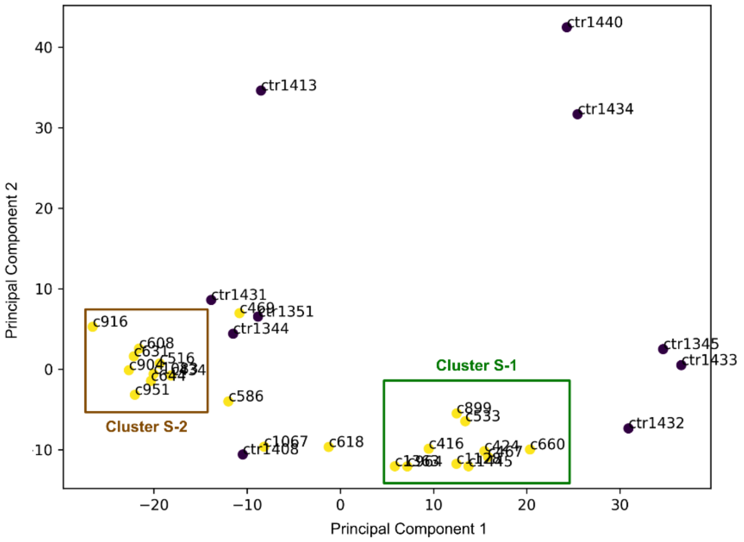

Supplemental Figure 4D. Hierarchical consensus clustering based on protein expression data of control and HGSOC samples **A)** Clustering matrices were obtained using ConsensusClusterPlus R package, fixing its analysis to a minimum of 2 clusters (k=2) or 6 subsets at maximum (k=6). The higher the consensus score was, the more likely they were assigned to the same group. Each colored bar in the upper part of each analysis defines a specific cluster. **B)** The cumulative distribution function (CDF) curves in consensus cluster analysis. CDF curves of consensus scores by different subtype numbers (*k* = 2, 3, 4, 5 and 6) were displayed **C)** Graphic depicting the cluster-consensus value of clusters at each k. This is the mean of all pairwise consensus values between a cluster's members. Cluster is indicated by color following the same color scheme as the clustering matrices. The bars are grouped by k, which is marked on the horizontal axis. High values indicate a cluster which has high stability and low values indicate a cluster has low stability.

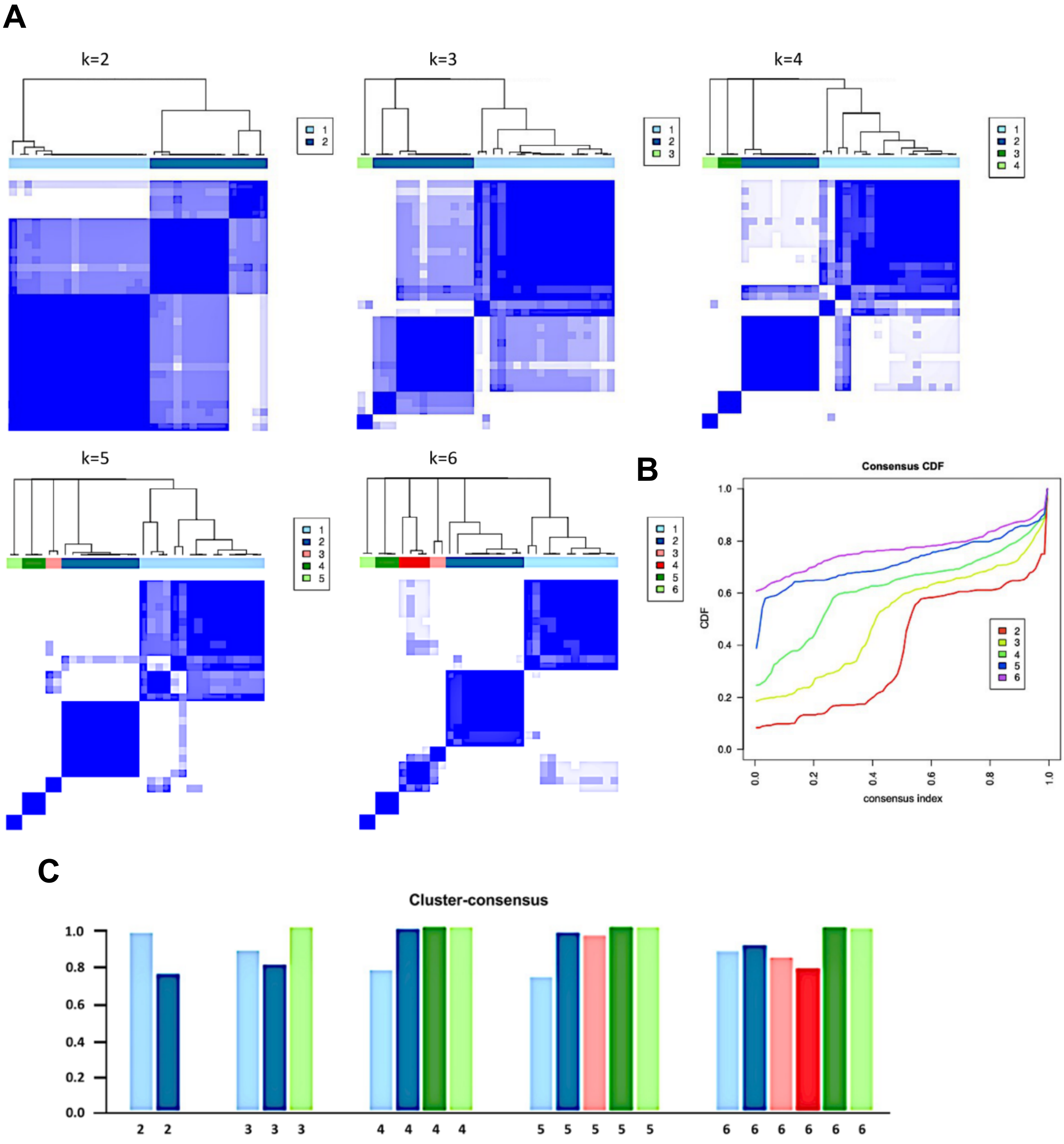

Supplemental Figure 5. Kaplan-Meier curve showing overall survival (OS) according to main proteomic HGSOC clusters (S1, n=3; S2, n=6). The analysis exclusively included patients with HGSOC who underwent primary or diagnostic surgeries and presented either mutations in susceptibility genes (BRCA1, BRCA2, or other genes related to HR) or defects in HR (HRD tumors).

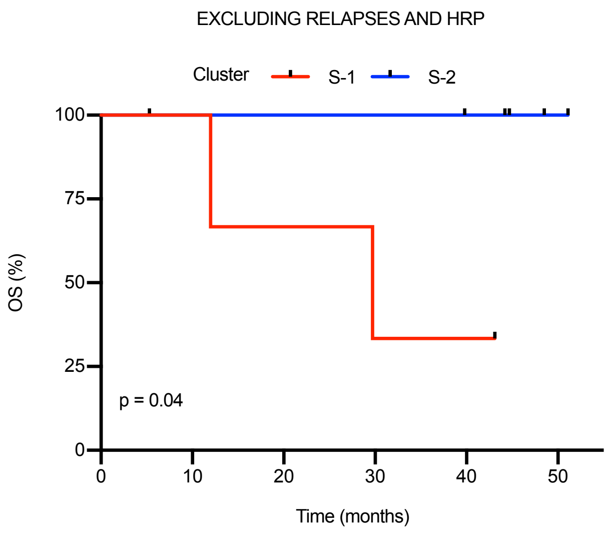

Supplemental Figure 6A. Correlation clustering map depicting the unsupervised analysis for HGSOC samples (n=22) and HRD status. The color bar on the left indicates the degree of correlation between two samples under study, with a value of 1 (dark red) indicating an identical sample in terms of protein cargo and -1 (dark blue) indicating potential samples with completely opposite profiles. On the right side of the figure, the main clusters (H1 and H2) are shown. Information is also provided regarding the presence of pathogenic alterations in BRCA1/2 loci (dark blue), in other HRD related susceptibility genes (PALB2, BRIP1, CCNE1, MYC and RAD21) (light blue boxes) or the presence of other molecular events suggestive of HR-deficient phenotypes (HRD+, light blue boxes). HR proficient tumors are depicted as white boxes.

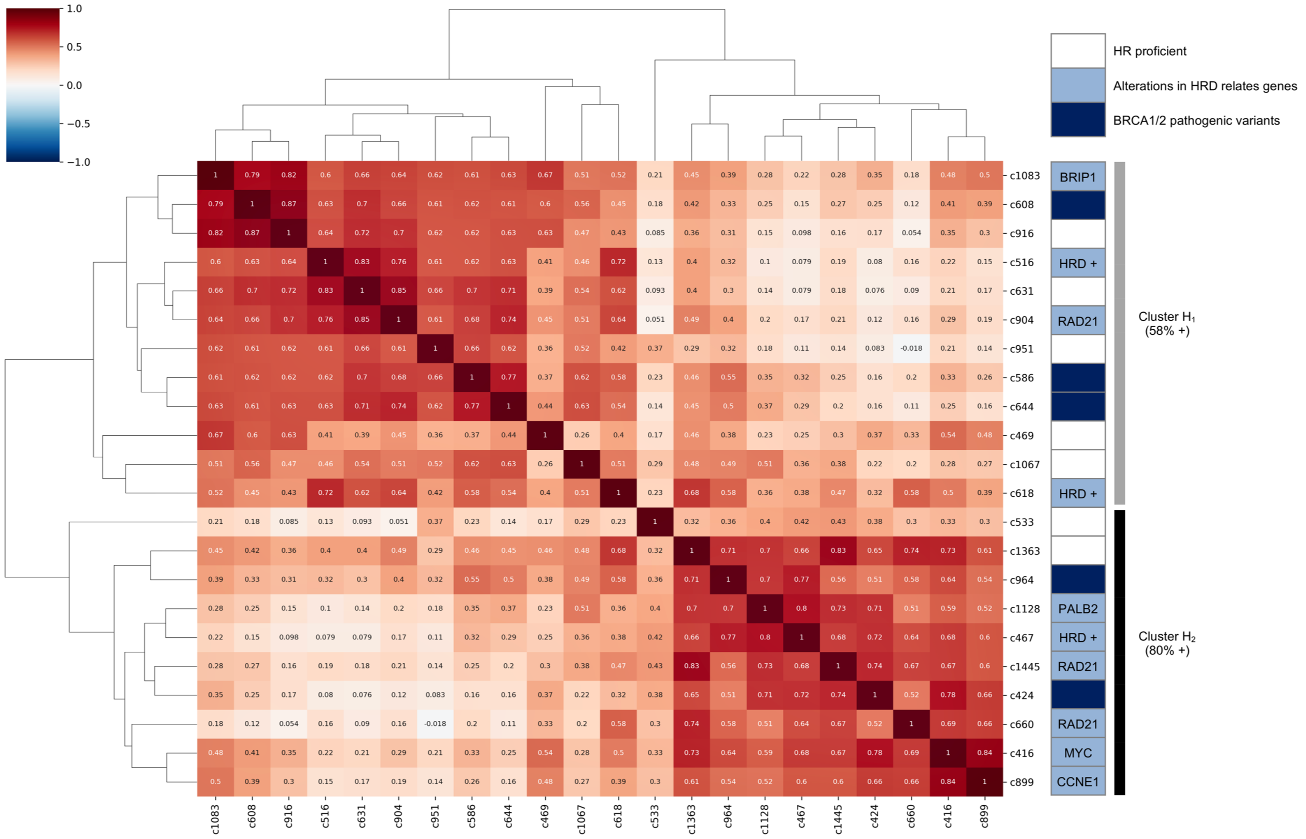

Supplemental Figure 6B. Correlation clustering map depicting the unsupervised analysis for HGSOC samples (n=18) and platinum-based sensitivity. The color bar on the left indicates the degree of correlation between two samples under study, with a value of 1 (dark red) indicating an identical sample in terms of protein cargo and -1 (dark blue) indicating potential samples with completely opposite profiles. On the right side of the figure, the main clusters (S-1 and S-2) are shown. Information is also provided on the response to platinum-based chemotherapy administered after sample collection: platinum-sensitive (green) and platinum-resistant (dark red).

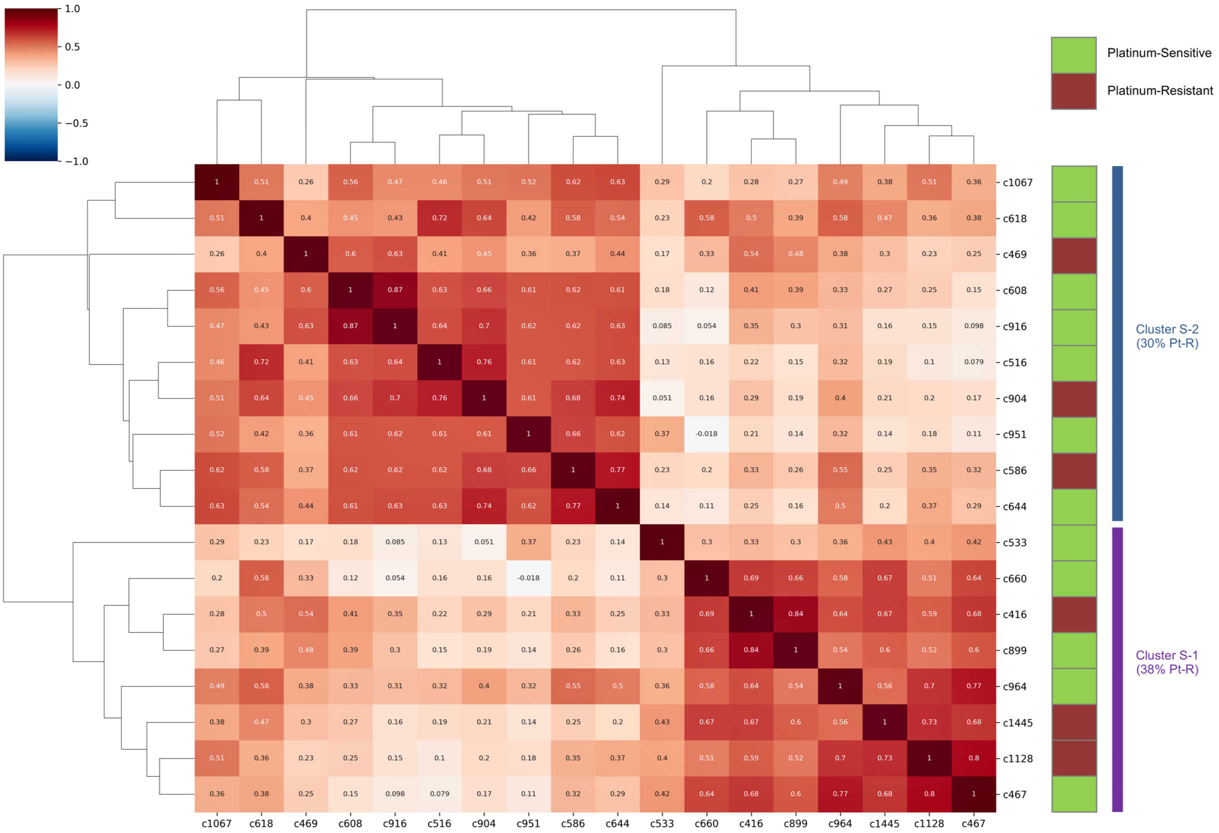

Supplemental Figure 7. (A) Images obtained from *in silico* String analysis, conducted to identify molecular interrelationships among significantly deregulated proteins (adjusted *p*-value≤0.05) with specific expression fold changes (FC, -2≤ FC ≤2) for HGSOC recurrences vs. primary/diagnostic comparison (differentially expressed proteins=181). **(B-C)** Graphs depicting proteomic data related to S100A4 and STX5 targets **(B)** Dot plot depicting individual S100A4 and STX5 z-scores in HGSOC (n≤23) vs. controls (Ct, n≤10). **(C)** S100A4 z-scores obtained through mass spectrometry profiling and categorized according to disease status, such as non-oncological specimens (controls, n=10), HGSOC primary samples (n=10), HGSOC interval surgeries (n=7) or HGSOC relapse samples (n=6). Data are represented by Median and IQR from each independent samples/experiments. *P*-values of significant findings (*, p-value<0.05; **, p-value<0.01 and ***, p-value<0.001, Mann-Whitney test [B] or Kruskal-Wallis test with Dunn's multiple comparison test and Bonferroni adjusted p-values [C]).

A

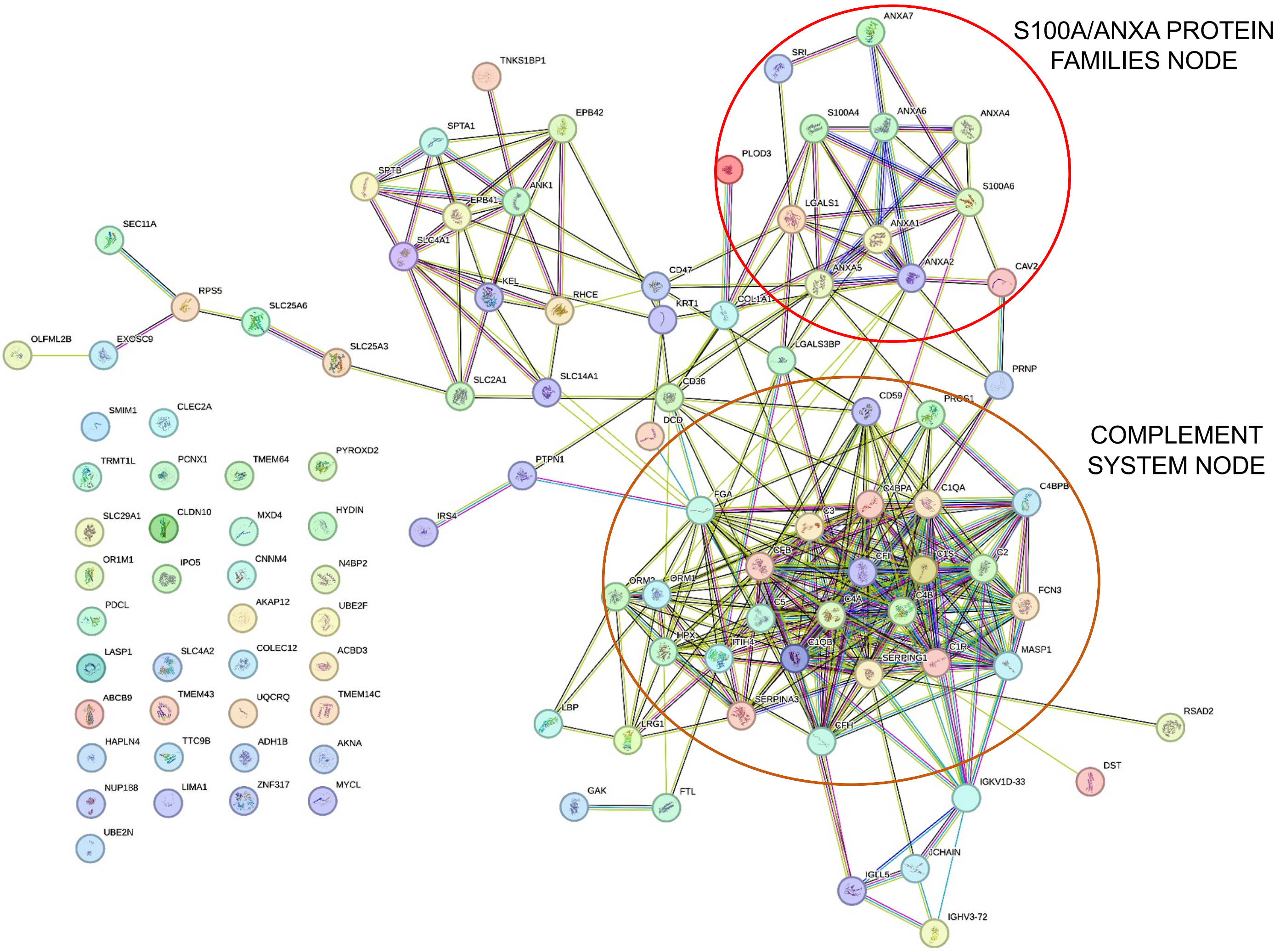

B

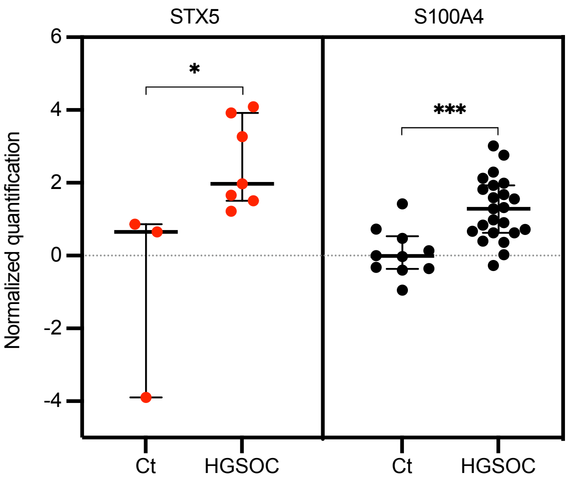
STX5
S100A4
6
4
2
0
-2
-4
Normalized quantification
*
***
Ct
HGSOC
Ct
HGSOC

C

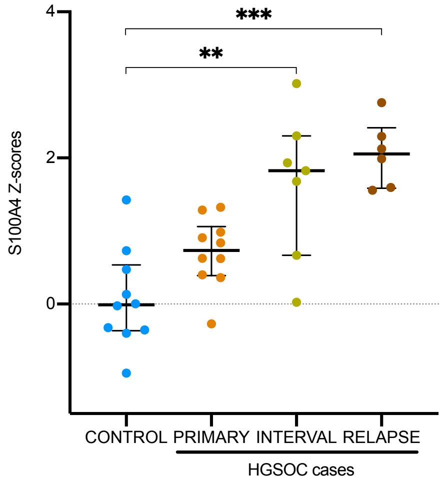
4
2
0
S100A4 Z-scores
***
**
CONTROL
PRIMARY
INTERVAL
RELAPSE
HGSOC cases

Supplemental figure 8. (A) Representative western blot images related to the concentration of S100A4, STX5 and CD9 proteins in samples from the validation cohort. The sample set tested by immunoblotting included 22 serous carcinomas and 5 controls. **(B)** S100A4/CD9 ratio obtained through immunoblotting in controls (CT, n=5) or HGSOC samples subclassified according to disease status (PRIM., primary/diagnostic [n=9]; INT., interval [n=8]; REL., relapse [n=5]). Western blot bands corresponding to the above-mentioned factors (S100A4 or CD9) were quantified using Image J software and the corresponding normalized ratio depicted in this graph as individual dots. Data are represented by Median and IQR from each independent samples/experiments. *P*-values of significant findings (*, p-value<0.05, Kruskal-Wallis test with Dunn's multiple comparison test and Bonferroni adjusted p-values [B]).

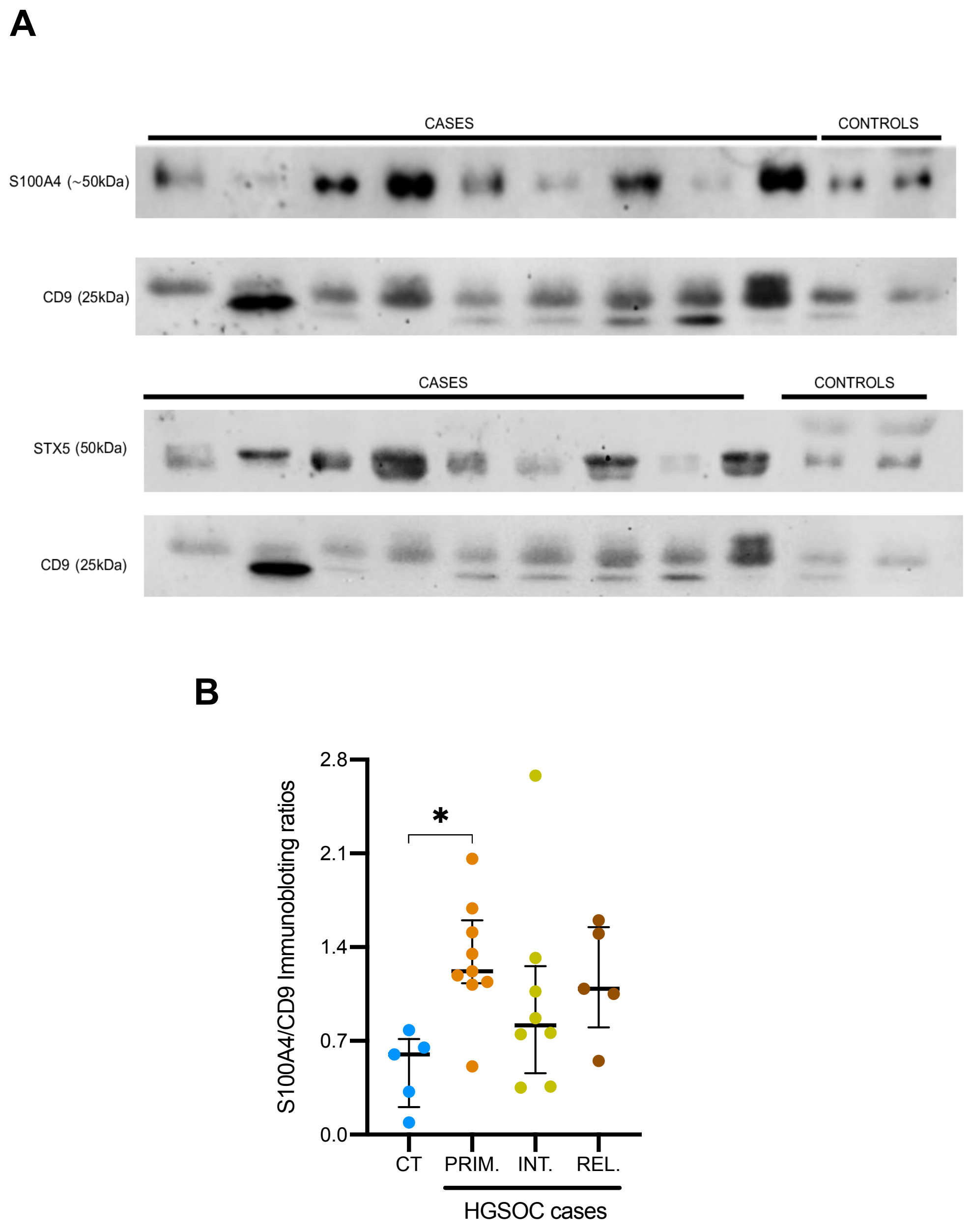

Supplemental Figure 9. Graphs and figures depicting biological processes and functional categories of interest enriched according to Enrichr package (7A-C) or GSEA (7D) in certain comparisons of interest. **(A)** Cellular compartments-related categories enriched in HGSOC compared to controls (adjusted *p*-value<0.01). **(B-C)** Categories included in Hallmark and Reactome enriched for the following comparisons (adjusted *p*-value<0.05): HGSOC vs. controls (B) or relapses vs. primary/diagnostic samples **(C)**. Relevant categories enriched for several comparisons are marked with an orange arrow (Hallmarks/Reactome). **(D)** GSEA-enhanced processes in molecular signatures databases similar to those previously used for Enrichr analysis.

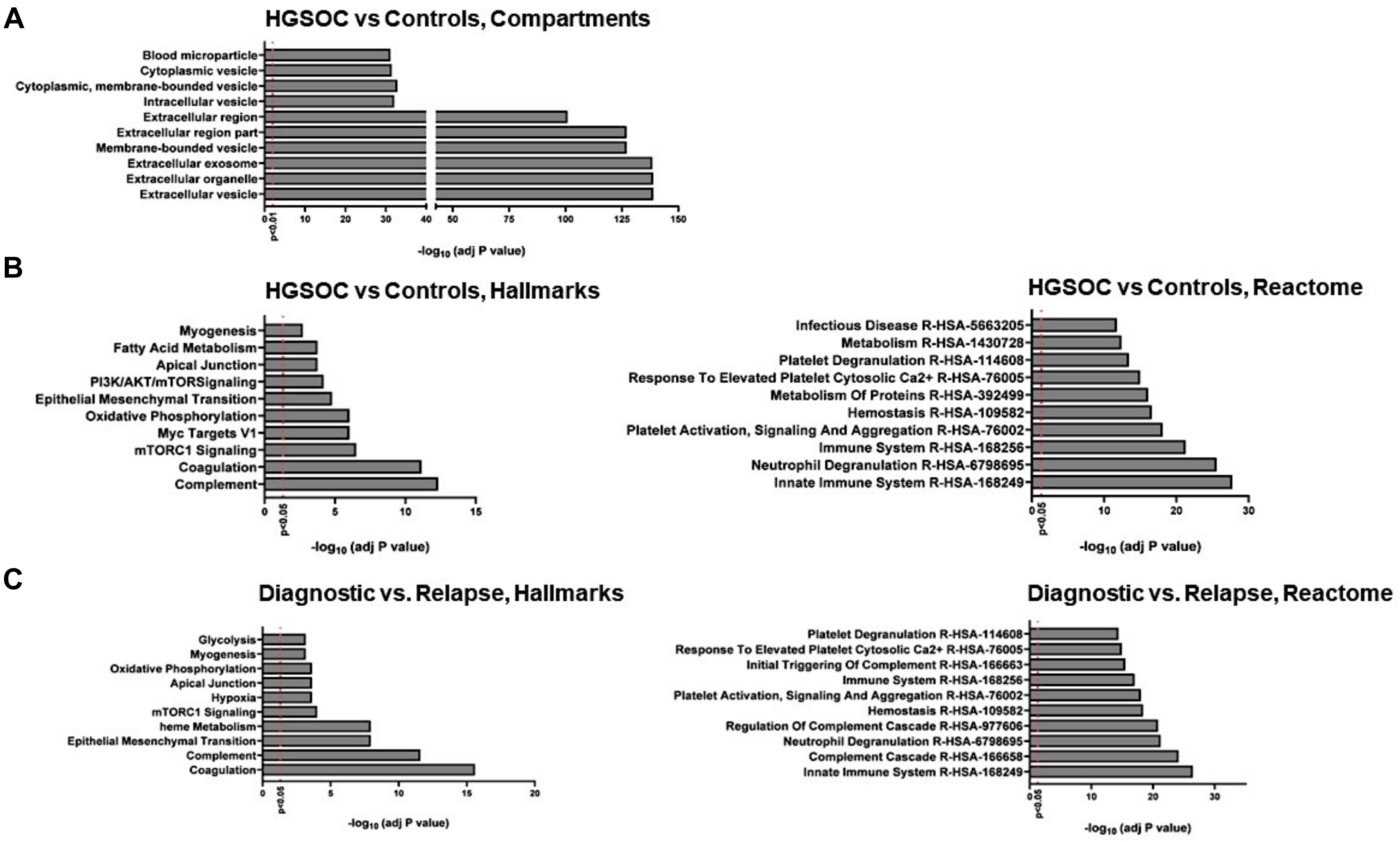

D

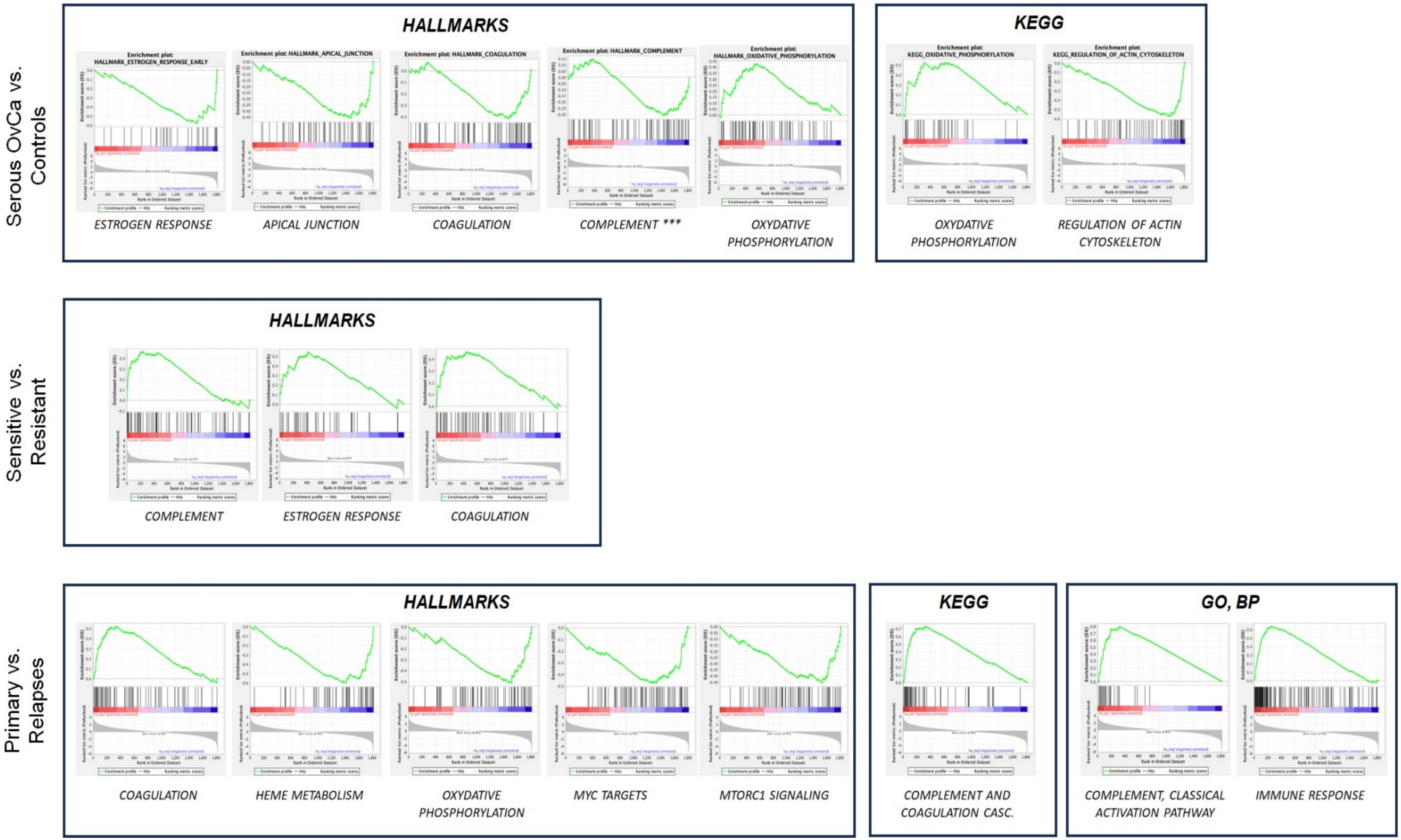

Serous OvCa vs. Controls
HALLMARKS
ESTROGEN RESPONSE
APICAL JUNCTION
COAGULATION
COMPLEMENT ***
OXYDATIVE PHOSPHORYLATION
KEGG
OXYDATIVE PHOSPHORYLATION
REGULATION OF ACTIN CYTOSKELETON
Sensitive vs. Resistant
HALLMARKS
COMPLEMENT
ESTROGEN RESPONSE
COAGULATION
Primary vs. Relapses
HALLMARKS
COAGULATION
HEME METABOLISM
OXYDATIVE PHOSPHORYLATION
MYC TARGETS
MTORC1 SIGNALING
KEGG
COMPLEMENT AND COAGULATION CASC.
GO, BP
COMPLEMENT, CLASSICAL ACTIVATION PATHWAY
IMMUNE RESPONSE

Supplemental Figure 10. Images depicting the data related to the correlation of proteomic data obtained from the S-1 vs. S-2 cluster comparison. **A)** PCA performed for the S-1 vs. S-2 comparison considering the PFD-sEVs proteomic data of the 25 most over- or under-contained proteins (shown in Table 3). **B)** Set of proteins contained in the PFD-sEVs of all HGSOC patients in our cohort and included in the prognostic transcriptional signature described by Yoshihara et al. (26). Proteins labelled in red color depicts those protein with a differential cargo between S1 vs. S2 clusters. logFC, fold-change logarithm; t, t statistic; adj.P.Val, adjusted p-value from Benjamini-Hochberg algorithm. **C)** Hallmark functional categories enriched in the set of 25 overcontained proteins in the S-1 cluster. **D)** Hallmark functional categories enriched in the set of 25 overcontained proteins in the S-2 cluster. Enrichment analysis was performed using *Enrichr in silico* tool.

A

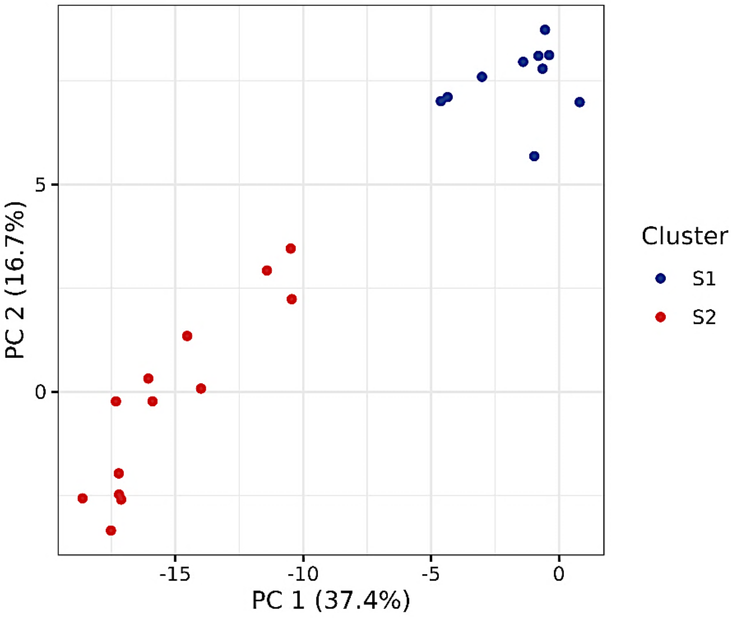

B

Protein	logFC	t	adj.P.Val
ANXA1	**-1.675**	-5.175	6.48E-05
SERPINA1	**1.438**	4.196	4.59E-04
APOL1	**1.238**	3.502	2.72E-03
ALOX5AP	**-1.213**	-3.450	3.16E-03
DSTN	**-1.154**	-3.263	5.04E-03
FCER1G	**0.879**	2.394	3.75E-02
PGK1	**-0.831**	-2.233	5.11E-02
HLA-DPB1	**0.442**	1.154	3.18E-01

C

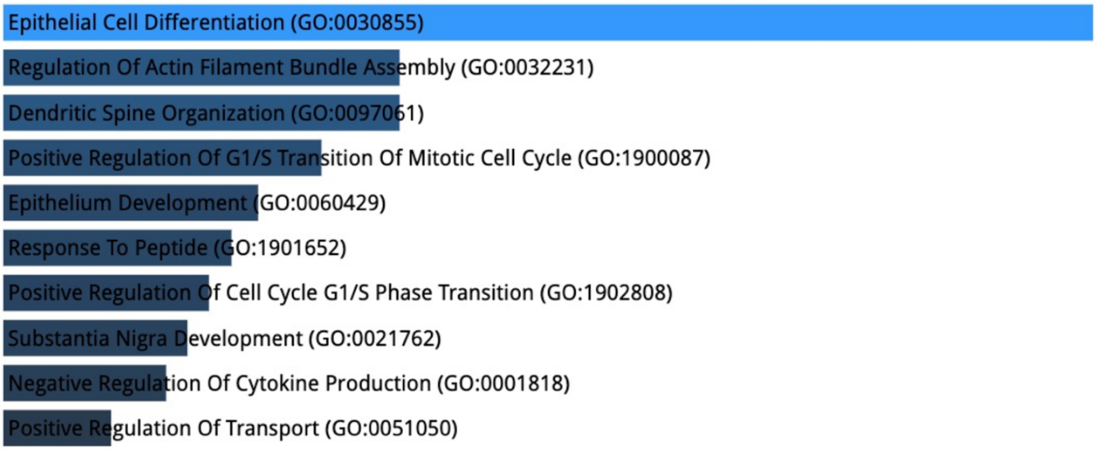
Epithelial Cell Differentiation (GO:0030855)
Regulation Of Actin Filament Bundle Assembly (GO:0032231)
Dendritic Spine Organization (GO:0097061)
Positive Regulation Of G1/S Transition Of Mitotic Cell Cycle (GO:1900087)
Epithelium Development (GO:0060429)
Response To Peptide (GO:1901652)
Positive Regulation Of Cell Cycle G1/S Phase Transition (GO:1902808)
Substantia Nigra Development (GO:0021762)
Negative Regulation Of Cytokine Production (GO:0001818)
Positive Regulation Of Transport (GO:0051050)

D

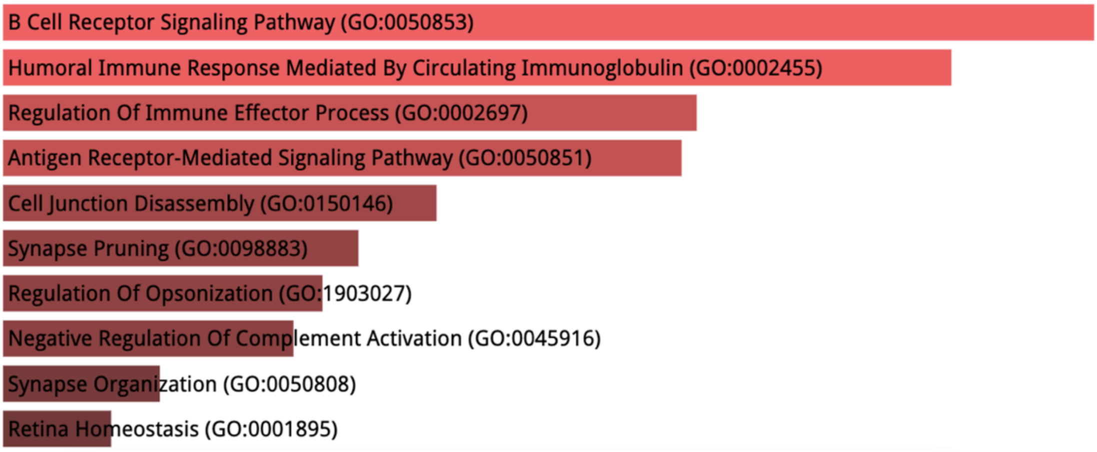
B Cell Receptor Signaling Pathway (GO:0050853)
Humoral Immune Response Mediated By Circulating Immunoglobulin (GO:0002455)
Regulation Of Immune Effector Process (GO:0002697)
Antigen Receptor-Mediated Signaling Pathway (GO:0050851)
Cell Junction Disassembly (GO:0150146)
Synapse Pruning (GO:0098883)
Regulation Of Opsonization (GO:1903027)
Negative Regulation Of Complement Activation (GO:0045916)
Synapse Organization (GO:0050808)
Retina Homeostasis (GO:0001895)
