# Supplementary material for: Proteomic profiles of peritoneal fluid-derived small extracellular vesicles correlate with patient outcome in ovarian cancer
Source: J Clin Invest. 2024 Apr 2;134(10):e176161. doi: 10.1172/JCI176161 (PMC11093605; doi:10.1172/JCI176161)
Supplement: Unedited blot and gel images [file jci-134-176161-s071.pdf]

Full unedited gel for
Figure 2D

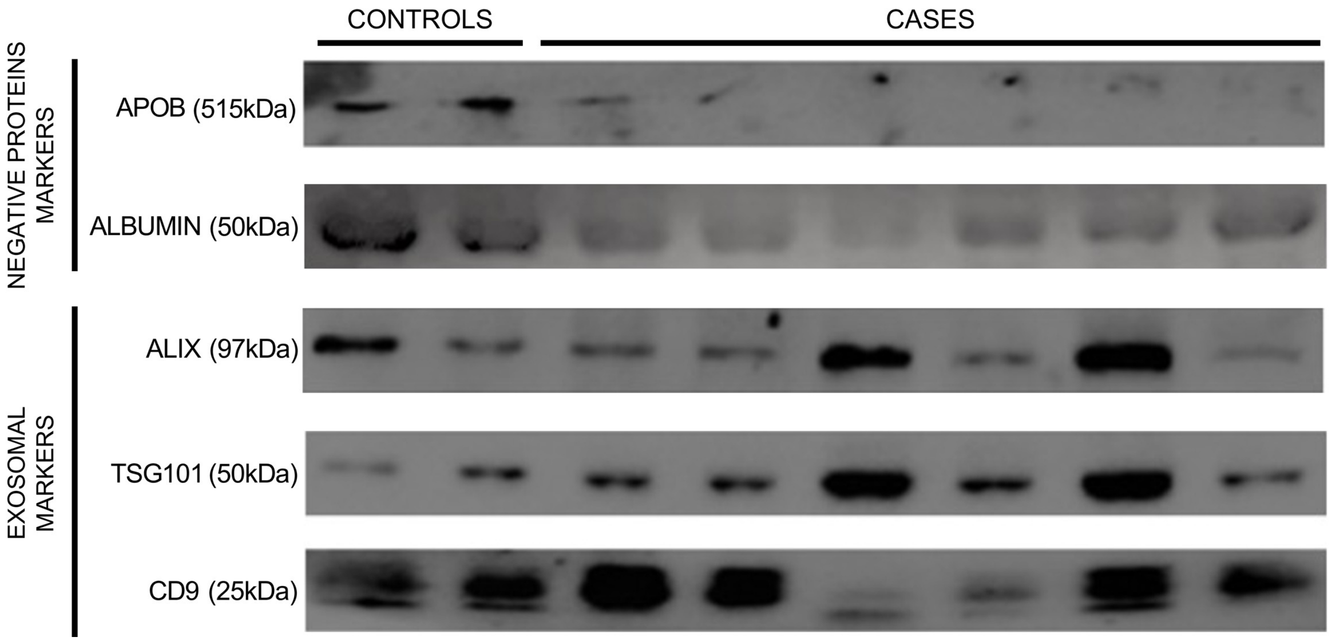
CONTROLS
CASES
NEGATIVE PROTEINS MARKERS
APOB (515kDa)
ALBUMIN (50kDa)
EXOSOMAL MARKERS
ALIX (97kDa)
TSG101 (50kDa)
CD9 (25kDa)

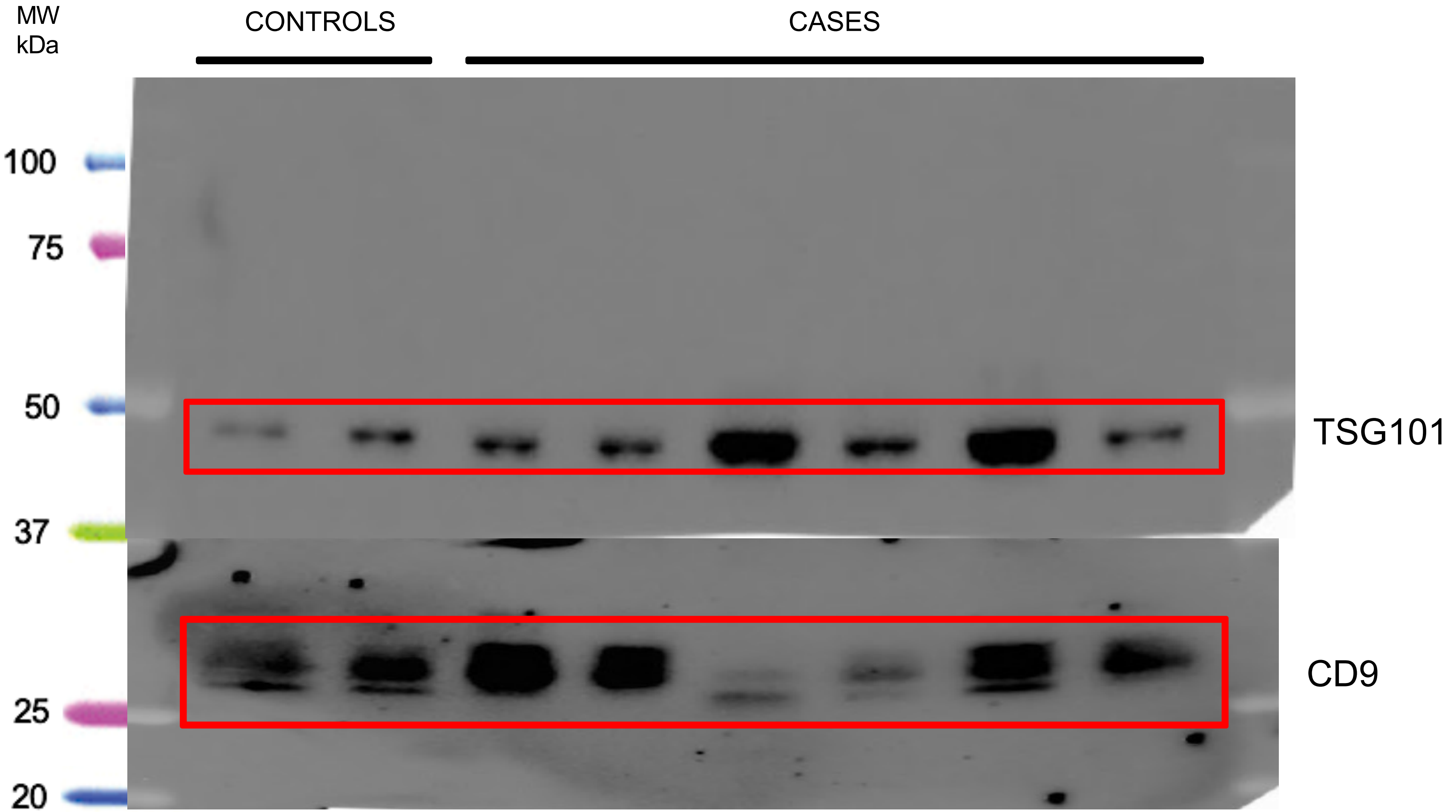
MW
kDa
CONTROLS
CASES
100
75
50
37
25
20
TSG101
CD9

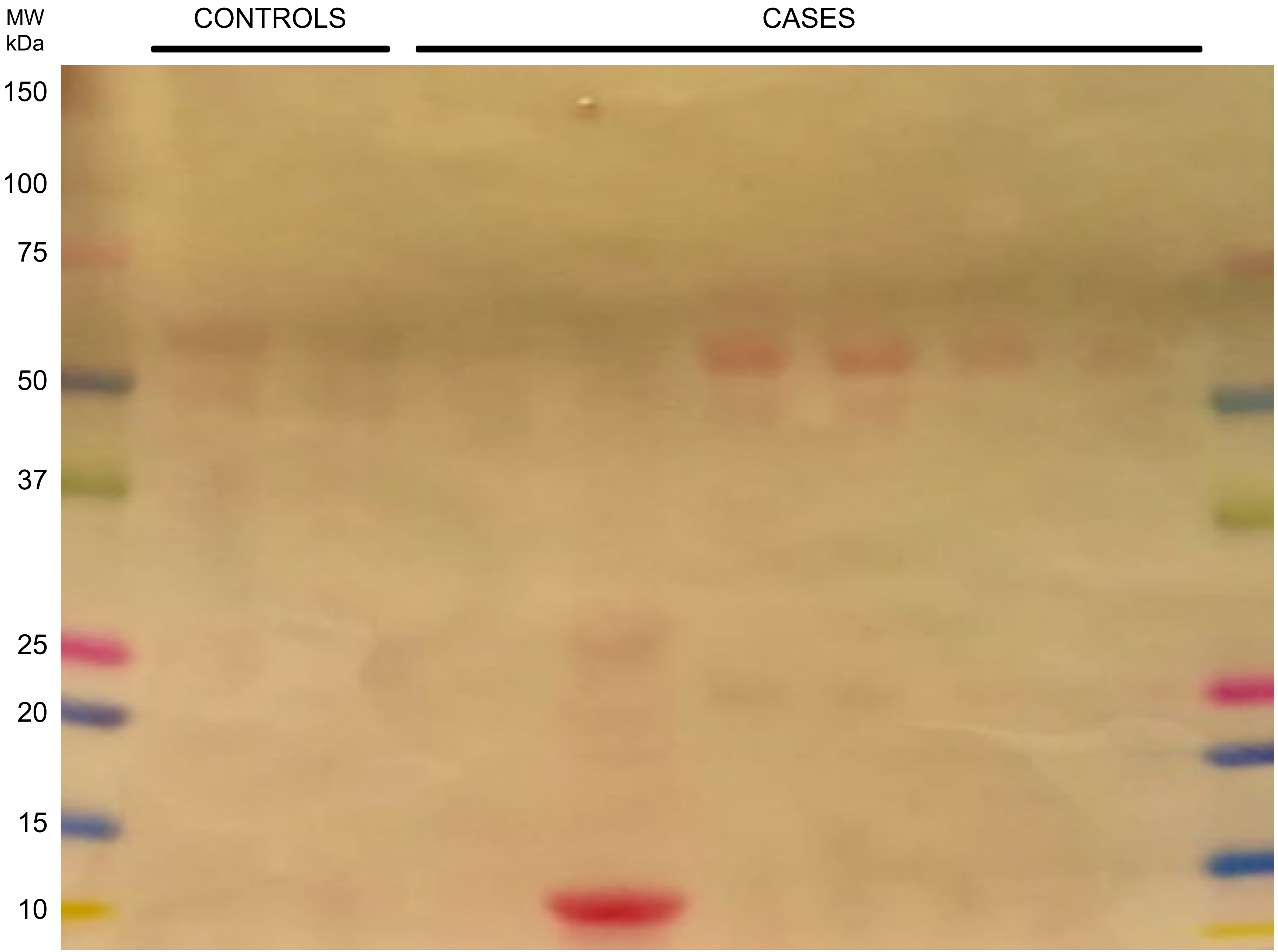
MW
kDa
CONTROLS
CASES
150
100
75
50
37
25
20
15
10

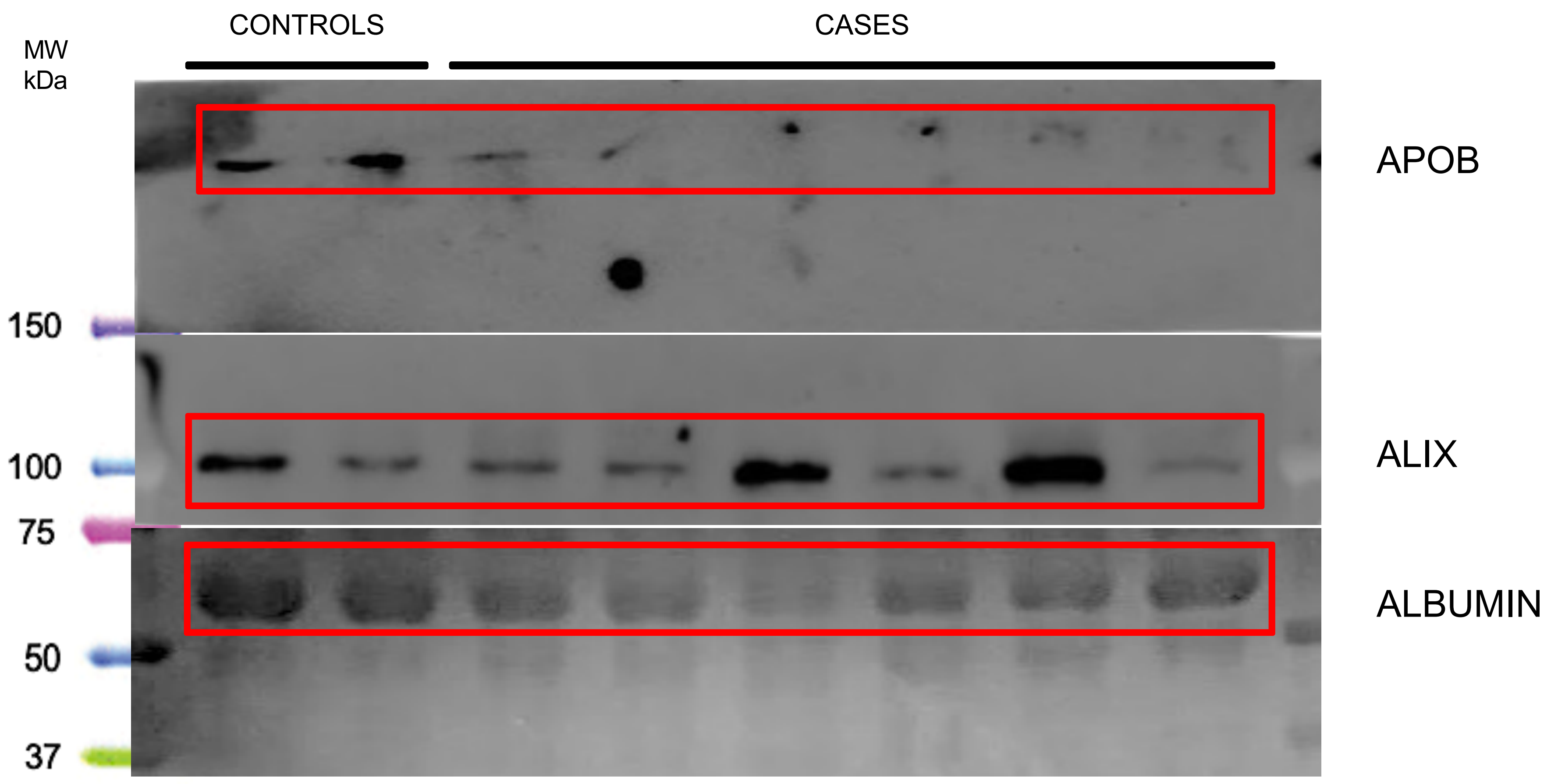

CONTROLS
CASES
MW
kDa
150
100
75
50
37
APOB
ALIX
ALBUMIN

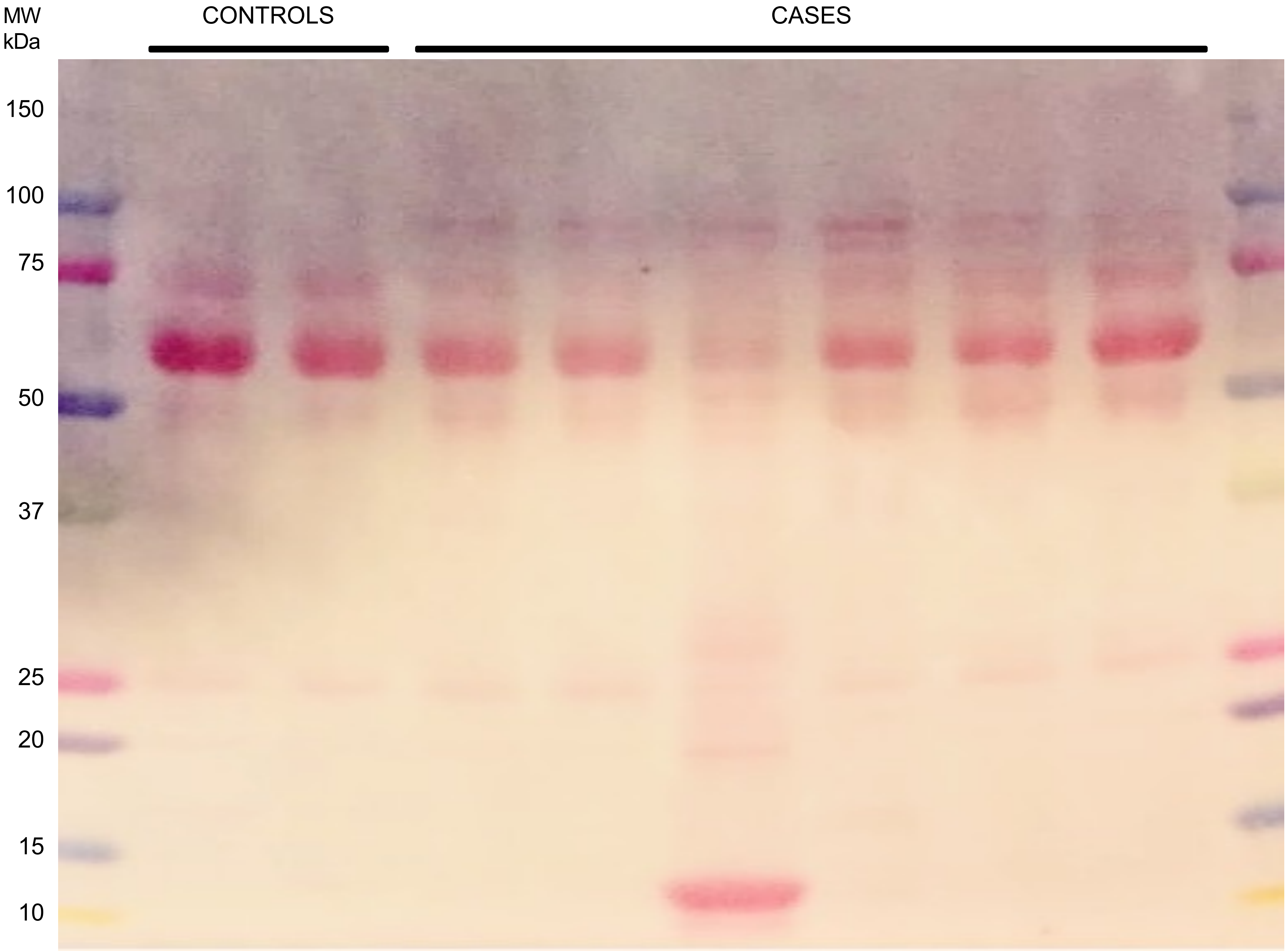
MW
kDa
CONTROLS
CASES
150
100
75
50
37
25
20
15
10

Full unedited gel for Figure 2E

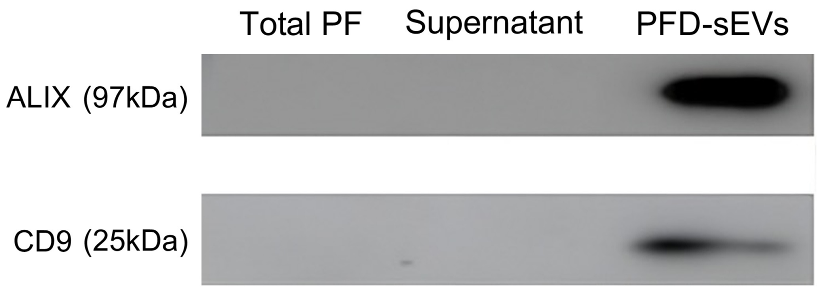
Total PF
Supernatant
PFD-sEVs
ALIX (97kDa)
CD9 (25kDa)

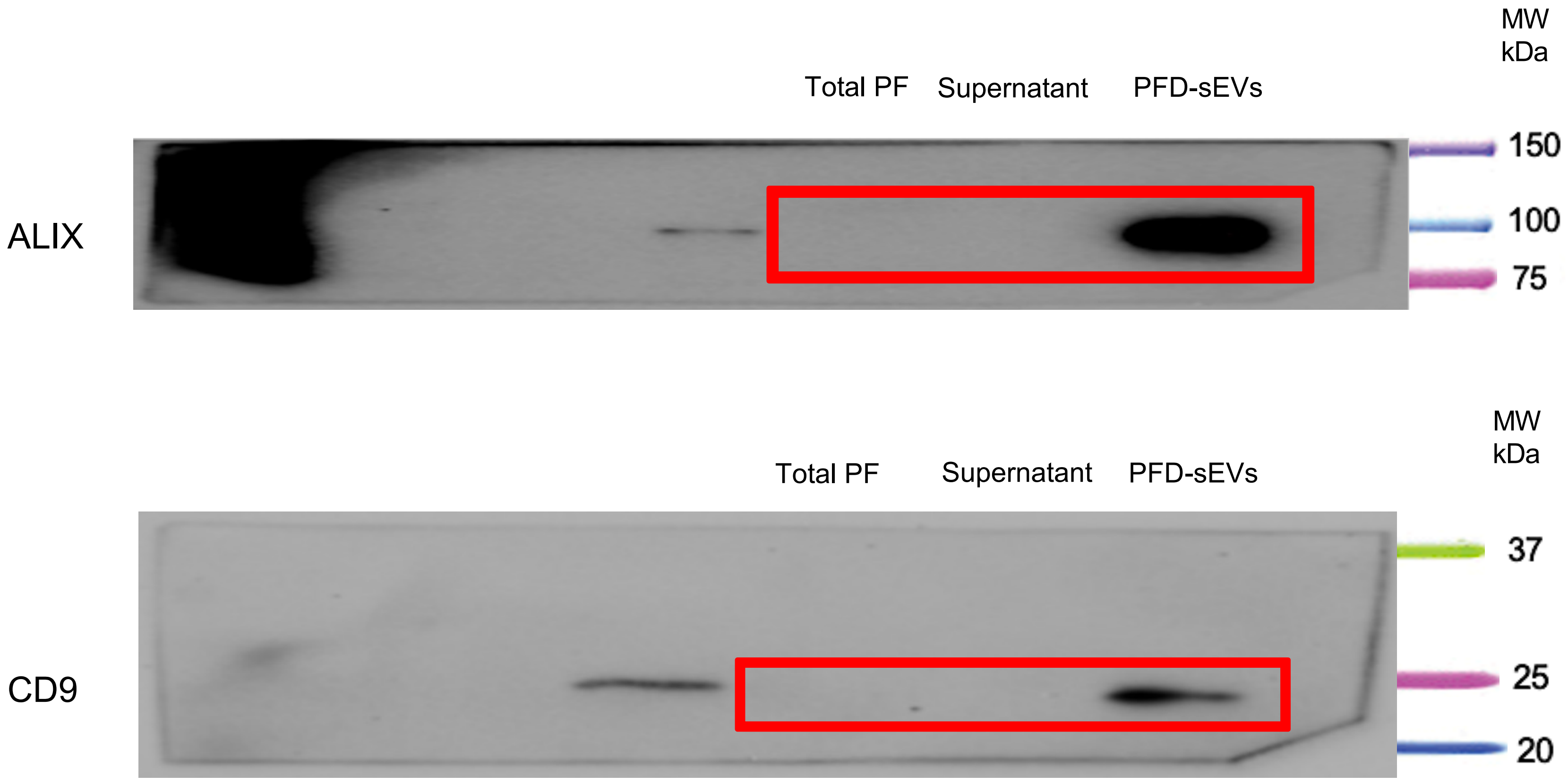

MW
kDa
Total PF
Supernatant
PFD-sEVs
150
100
75
ALIX
MW
kDa
Total PF
Supernatant
PFD-sEVs
37
25
20
CD9

Full unedited gel for Supplemental Figure 2E

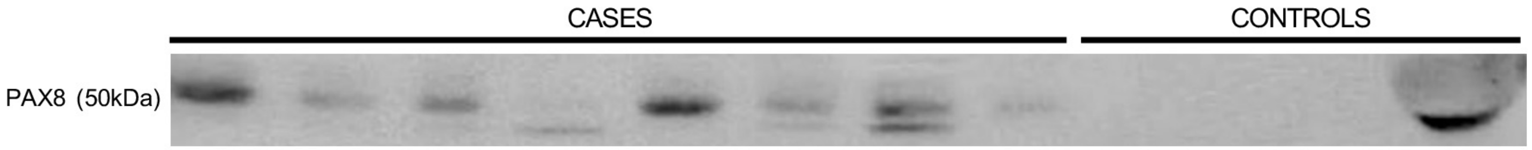
CASES
CONTROLS
PAX8 (50kDa)

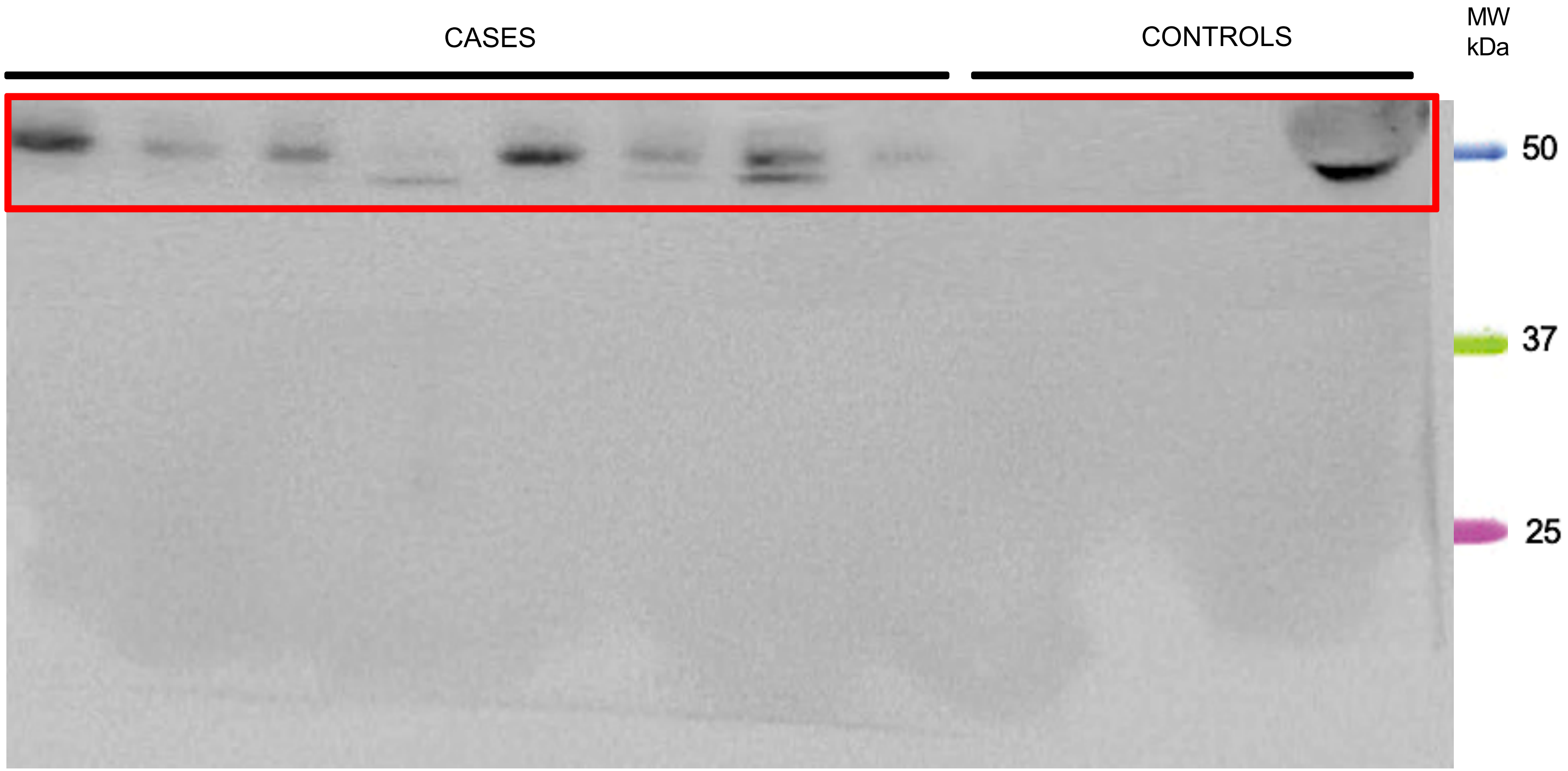
CASES
CONTROLS
MW
kDa
50
37
25

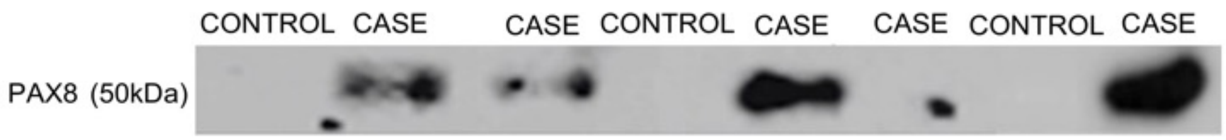
CONTROL
CASE
CASE
CONTROL
CASE
CASE
CONTROL
CASE
PAX8 (50kDa)

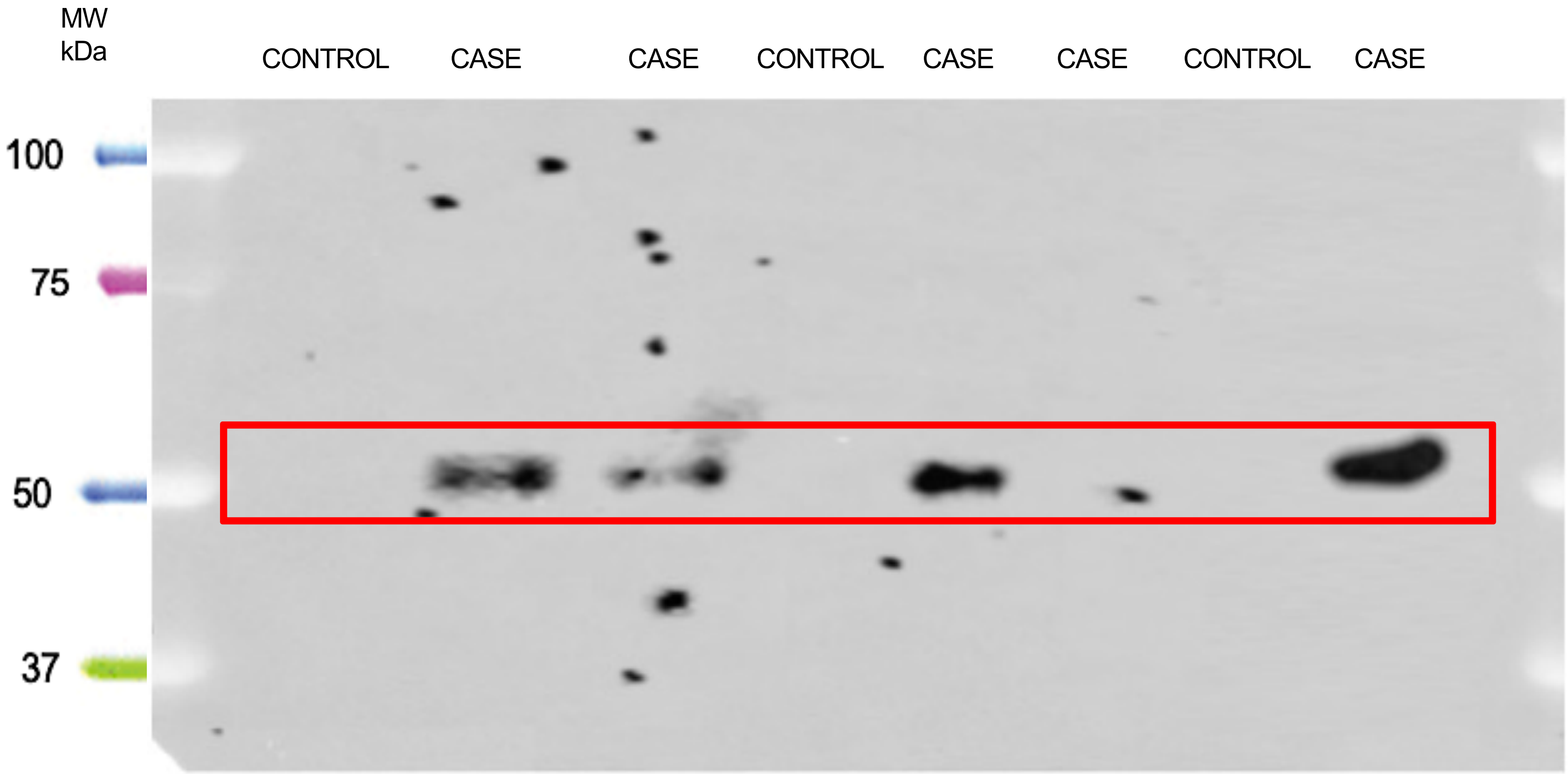
MW
kDa
CONTROL
CASE
CASE
CONTROL
CASE
CASE
CONTROL
CASE
100
75
50
37

Full unedited gel for Supplemental Figure 8A

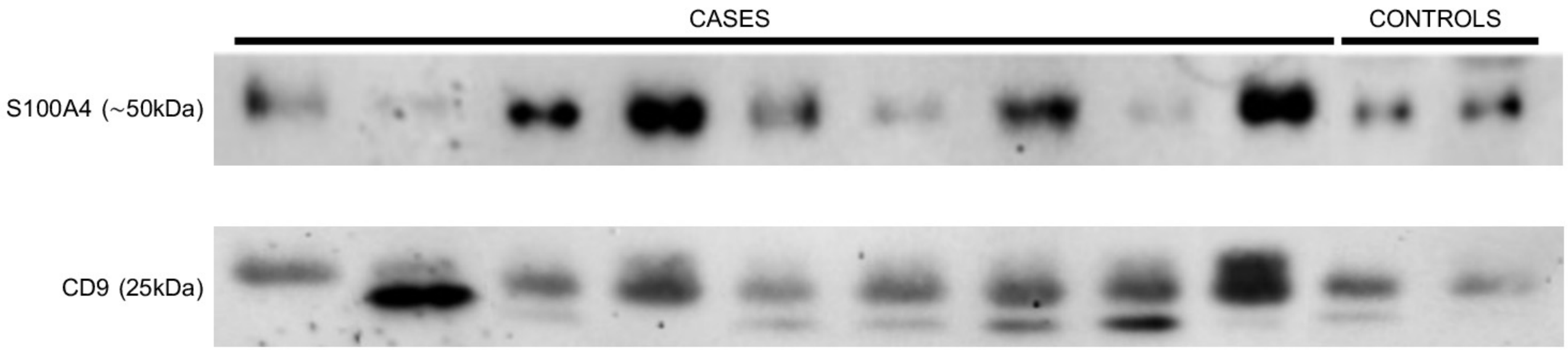
CASES
CONTROLS
S100A4 (~50kDa)
CD9 (25kDa)

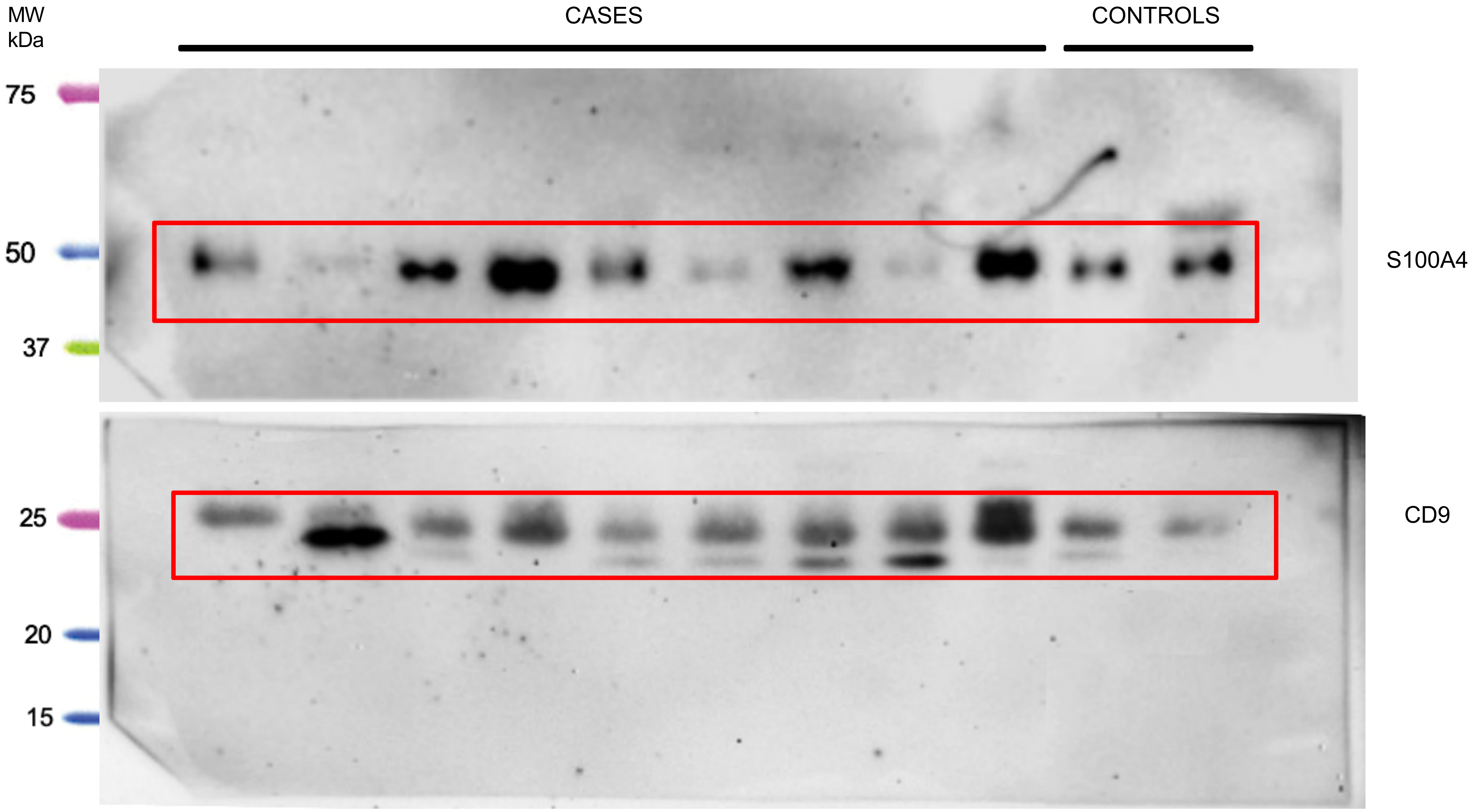
MW
kDa
CASES
CONTROLS
75
50
37
S100A4
25
20
15
CD9

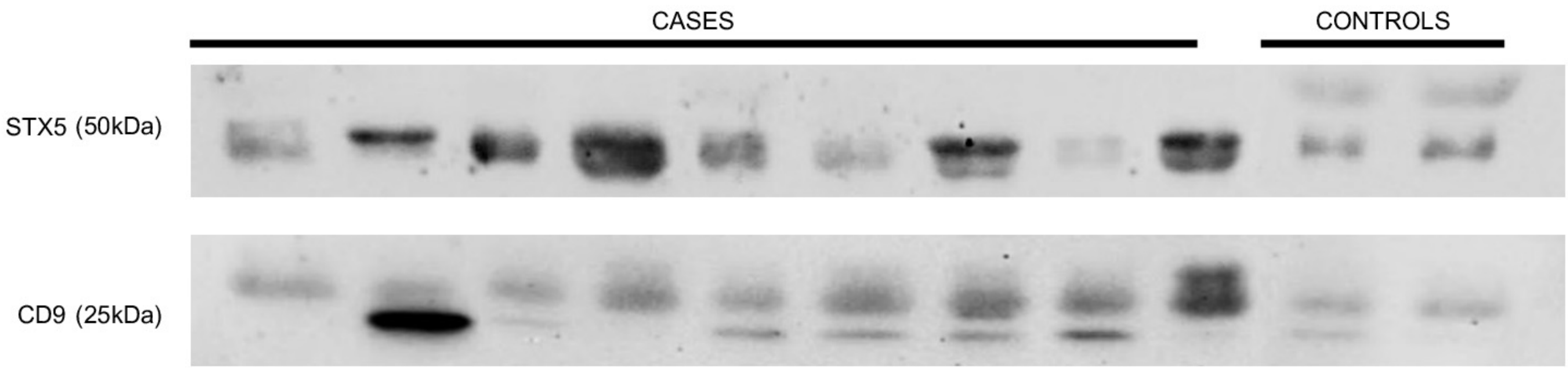
CASES
CONTROLS
STX5 (50kDa)
CD9 (25kDa)

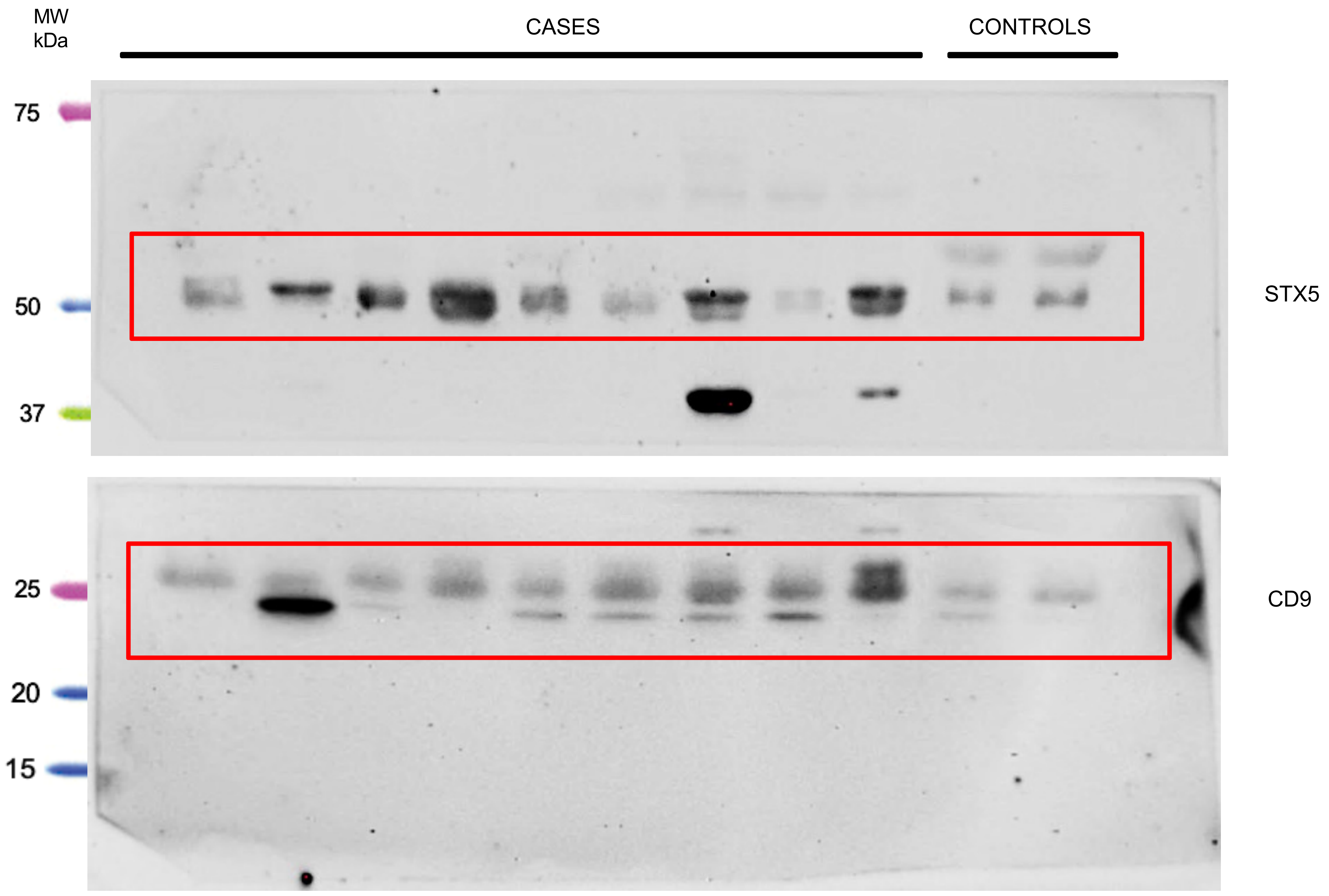
MW
kDa
CASES
CONTROLS
75
50
37
STX5
25
20
15
CD9
